# Regulation of Aromatic Compounds and Environmental Stimuli Response by the MarR Family Regulator AesR in *Corynebacterium glutamicum*

**DOI:** 10.3390/microorganisms14071416

**Published:** 2026-06-28

**Authors:** Meiru Si, Qimiao Shi, Meng Shao, Shuli Wang, Runge Xu, Ruixue Wang, Tao Su, Can Chen

**Affiliations:** 1 Shandong Key Laboratory of Wetland Ecology and Biodiversity Conservation in the Lower Yellow River, College of Life Sciences, Qufu Normal University, Qufu 273165, China; 2 Key Laboratory of Plant Genetics and Molecular Breeding, College of Life Science and Agronomy, Zhoukou Normal University, Zhoukou 466001, China

**Keywords:** MarR-type regulator, *C. glutamicum*, aromatic compounds, stress response, antibiotics

## Abstract

The MarR family regulators, widespread in bacteria and archaea, control diverse cellular processes, yet the regulatory mode and molecular signaling mechanism remain unclear in *Corynebacterium glutamicum*. Here, we functionally characterize AesR (aromatic compounds and environmental stimuli-sensing regulator), a MarR-type transcriptional regulator encoded by *ncgl0019* in *C*. *glutamicum*. RNA sequencing (RNA-seq) analysis of an *aesR*-deleted strain (Δ*aesR*) revealed the down-regulation of genes involved in aromatic compounds degradation, stress response, antibiotic resistance and cell envelope biogenesis, correlating with heightened sensitivity of Δ*aesR* to adverse conditions. RNA-seq, quantitative reverse transcription-PCR (qRT-PCR) and promoter activity analysis uncovered that AesR represses its own operon (including the Zn-dependent protease with chaperone function gene *ncgl0020*) and the divergent cytochrome C biosynthesis operon *ncgl0018*-*ncgl0017*. AesR binds as a dimer to two side-by-side inverted repeats [5′-ACTATG-N_3_-CATAGT*CGACTA*-N_7_-*TAGTTG*-3′] in the *ncgl0018-aesR* intergenic region with different affinity, and Cu^2+^/Ni^2+^/Zn^2+^ disrupted binding. These metal ions, along with aromatic compounds, organic peroxides, and bactericidal antibiotics, induce both operons in vivo. Notably, penicillin elevates intracellular Cu^2+^/Ni^2+^/Zn^2+^ levels. Collectively, our findings identify AesR as a novel regulator that senses metal ions as direct signals, relieving autorepression and enabling bacterial defense against aromatic compounds and environmental stressors.

## 1. Introduction

Lignocellulose represents a highly abundant renewable resource for manufacturing biofuels and biobased chemicals. However, during the pretreatment of lignocellulosic biomass, various inhibitory compounds—particularly aromatic compounds—are generated. These compounds exert toxic effects on microorganisms, significantly impairing microbial fermentation and the synthesis of target products. Therefore, fully elucidating the mechanisms by which fermentation-adapted microbes tolerate aromatic compounds is crucial for engineering resilient strains with resistance to lignocellulosic hydrolysate-derived inhibitors [1].

Notably, *Corynebacterium glutamicum* has recently been shown to use diverse lignin-derived aromatic compounds [such as ferulic acid (FA), vanillin, phenol, benzoate, phenylacetic acid, and 4-cresol] as its sole carbon and energy source for growth [1]. Its exceptional ability to degrade phenolic compounds and convert them into usable carbon sources gives *C. glutamicum* a distinct edge in industrial fermentation using lignocellulosic hydrolysates. Systematic, in-depth studying the regulatory genes related to the degradation and tolerance of aromatic compound inhibitors, combined with targeted genetic engineering for *C. glutamicum*, offers a highly promising strategy for developing efficient industrial strains capable of utilizing renewable lignocellulosic biomass.

*Corynebacterium glutamicum* shows strong adaptability to various adverse stresses encountered during fermentation, mainly by activating antioxidant defense systems. These systems include the synthesis of mycothiol (MSH) and the expression of a range of protective proteins, such as transcriptional regulatory proteins [2,3,4]. Based on the genome sequence, *C. glutamicum* had more than three hundred predicted transcriptional regulators grouped in several families, including the MarR (multiple antibiotic resistance regulator) family with at least nine members. The MarR family regulators are well-known sensors for controlling much more numerous and diverse physiological functions, including the catabolism of a variety of aromatic compounds, multidrug resistance, stress responses, central metabolism, and virulence factor production [5]. So far, three substrate metabolism-involving transcriptional regulators (the *malic* regulator MalR, phenylpropanoid regulator PhdR, and geranylgeranyl diphosphate regulator CrtR) [6,7,8] and four thiol-based redox-sensing sensors have been characterized in *C. glutamicum*. However, the natural inducers (real signals) and the regulatory molecular mechanisms of protein–ligand interactions for many MarR homologues from *C. glutamicum* remain unknown. Thus, an in-depth study of the regulatory mechanism and binding selectivity of MarR proteins to its inducer and promoter can lead to a more profound understanding of how bacteria build adaptive responses through MFTFs (MarR family of transcription factors).

In this study, we designated the *ncgl0019* gene, which encodes a putative MarR-family transcriptional regulator, as *aesR* (aromatic compounds and environmental stimuli-sensing regulator) based on experimental findings; the encoded protein is henceforth termed AesR. This gene constitutes an operon alongside *ncgl0020*, which putatively encodes a putative Zn-dependent protease, and is divergently oriented with respect to the *ncgl0018*-*ncgl0017* operon encoding the putative cytochrome C (CytC) biogenesis proteins. The majority of characterized MarR proteins are transcriptional repressors, and their genes are generally found adjacent to or part of the regulated gene cluster [9]. The genetic organization of AesR allowed us to investigate the function of *C. glutamicum* AesR in response to environmental stresses, including lignocellulose-derived aromatic compound inhibitors. Herein we reported that AesR was shown to repress *ncgl0018*-*ncgl0017*, *aesR*-*ncgl0020* and *ncgl2421* gene. AesR directly bound to an intergenic region between *ncgl0018*-*ncgl0017* and *aesR*-*ncgl0020* overlapping with the promoter elements of both. The in vitro DNA binding activity of AesR was inhibited by Cu^2+^/Ni^2+^/Zn^2+^. *aesR* mutant showed a decreased resistance to aromatic compounds, bactericidal antibiotics, Cu^2+^, Ni^2+^, and organic hydroperoxides (OHPs). The physiological functions of AesR in defending against various adverse stresses were further validated by its induced expression under multiple stress conditions. Penicillin (PEN) as a bactericidal antibiotic was found to increase the level of intracellular Cu^2+^/Ni^2+^/Zn^2+^, indicating that this Cu^2+^/Ni^2+^/Zn^2+^-triggered AesR derepression acted as the underlying mechanism for PEN-mediated derepression of *ncgl0018*-*ncgl0017* and *aesR*-*ncgl0020* inside *C. glutamicum* and subsequently AesR activated the target genes to promote bacterial resistance to the adverse stimuli.

## 2. Materials and Methods

### 2.1. Bacterial Strains and Culture Conditions

The bacterial strains and plasmids used in this study were listed in Table S1. All cloning was performed in *E. coli* JM109 (Stratagene, La Jolla, CA USA). *E. coli* and *C. glutamicum* were cultivated at 37 °C and 30 °C in either lysogeny broth (LB) aerobically on a rotary shaker (220 rpm) or on LB agar plates as previously reported, respectively [10]. For the generation of mutants and maintenance of *C. glutamicum*, brain heart infusion containing 0.5 M sorbitol (BHIS) medium was used [10]. Mineral salt (MS) medium with glucose (Glc) and/or various agents (antibiotics, aromatic compounds, metal ions) was used for expression analysis [10]. The Δ*aesR* and Δ*ncgl0020* mutants of *C. glutamicum* were achieved using the knockout vectors pK18*mobsacB*-Δ*aesR* and pK18*mobsacB*-Δ*ncgl0020* via a two-step homologous recombination protocol as described previously, respectively [11]. Detailed procedures for mutant construction are provided in the Supplementary Methods. The amount of antibiotics added follows the previously described method [12].

### 2.2. Plasmid Construction

Primers used in this study are listed in Table S2. Plasmid purification, DNA manipulation, and agarose gel electrophoresis were performed according to previously established protocols [12].

### 2.3. Protein Expression and Purification

Protein expression and purification were performed according to our previously established methods [13]. *E. coli* BL21 (DE3) host cells (Stratagene, La Jolla, CA, USA) transformed with pET28a-*aesR*, pET28a-*ncgl0020*, or pET28a-SUMO-*aesR* derivatives were grown at 37 °C in 800 mL LB medium with 50 µg/mL Kan to an OD_600_ of 0.4 before adding 0.1 mM IPTG for induction of the gene expression. After adding IPTG, the cultures were incubated at 20 °C for additional 8 h. After cells were harvested by centrifugation, His_6_-AesR, His_6_-NCgl0020 and His_6_-SUMO-AesR were purified by nickel affinity chromatography using nickel-activated nitrilotriacetic acid-agarose (NTA) (Novagen, San Diego, CA, USA) according to manufacturer’s instructions. Cleavage of the His_6_-SUMO tag was performed by adding PUP1 (0.04 mg PUP1 protein was used to cut 1 mg His_6_-SUMO-AesR) and incubation at 4 °C overnight. Ni-NTA resin was used to remove the cleaved tag and uncleaved protein from the His_6_-SUMO-free protein. Recombinant proteins obtained were dialyzed against the appropriate buffer overnight at 4 °C and stored at −80 °C until use. The purity of the purified protein was verified as >95% homogeneity based on sodium dodecyl sulfate polyacrylamide gel electrophoresis (SDS-PAGE) analysis. Protein concentrations were determined with the Bradford assay kit (Bio-Rad Laboratories, Hercules, California, USA) using bovine serum albumin (BSA) as reference.

### 2.4. Sensitivity Assays

Sensitivity assays were conducted by measuring bacterial growth in LB medium supplemented with varying concentrations of diverse agents, following the method described by Helbig et al. [14].

To measure the response to various aromatic compounds, overnight cultures of the corresponding *C. glutamicum* strains grown in LB medium at 30 °C were diluted 100-fold with LB medium, and the diluted cells were exposed to different concentrations of various aromatic compounds at 30 °C with shaking. After 30 min treatment, each sample (100 μL) of the 1:10,000 dilution was spread evenly over a fresh LB agar plate, and the colonies were counted after 48 h of incubation at 30 °C. Percentage survival was calculated as follows: [(CFU (Colony-Forming Units) mL^−1^ after challenge under different stresses)/(CFU mL^−1^ without stress challenge)] × 100.

Stationary phase *C. glutamicum* strains grown in LB medium were collected, washed, and diluted 30-fold into phosphate-buffered saline (PBS) containing 1 μM metal ions or 1 mM chelator ethylene diamine tetraacetic acid (EDTA), and then treated with 2.5 mg/mL PEN and 320 μg/mL erythromycin (ERY) 30 °C for 30 min. After treatment, the cultures were serially diluted and plated onto LB agar plates, and colonies were counted after 48 h growth at 30 °C. The percentage survival was calculated by dividing number of CFU of stressed cells by number of CFU of cells without stress.

### 2.5. Size Exclusion Chromatography

For determination of the molecular weight by size exclusion chromatography, we followed the previous method [13].

### 2.6. DNase I Footprinting Assay

DNase I footprinting was conducted in accordance with the method reported by Liu et al. [15].

### 2.7. Western Blot Analysis

Western blot analysis was conducted in accordance with previously reported protocols [3]. Primary antibodies at 4 °C overnight: anti-NCgl0018 rabbit polyclonal antibody, 1:1000; anti-NCgl0020 rabbit polyclonal antibody, 1:1000; anti-CytC rabbit polyclonal antibody, 1:1000 (ABclonal, Boston, MA, USA); anti-cytosolic RNA polymerase α (α-RNAP), 1:5000 (BioLegend Way, San Diego, CA, USA). The α-RNAP was used as a loading control. The anti-NCgl0018 and anti-NCgl0020 rabbit polyclonal antibodies were generated and affinity-purified according to the method described previously [3]. The density of bands on Western blots was quantified by Image Lab (Bio-Rad, Hercules, CA, USA).

### 2.8. RNA Sequencing (RNA-Seq)

The RNA-seq experiment was performed in accordance with our previously established protocol [16]. Total RNA was extracted from the exponentially growing *C. glutamicum* RES167 parental strain and ∆*aesR* mutant (3 biological replicates) via the RNeasy Mini Kit (Qiagen, Hilden, Germany) and the DNase I Kit (Sigma-Aldrich, Taufkirchen, Germany). RNA degradation and contamination were monitored on 1% agarose gels; RNA purity was checked using a NanoPhotometer spectrophotometer (IMPLEN, Westlake Village, CA, USA), and RNA integrity was assessed using a Bioanalyzer 2100 system (Agilent Technologies, Santa Clara, CA, USA). A total of 5 µg RNA per sample was used as input material in RNA sample preparations for subsequent cDNA library construction. All 6 samples had RIN values above 7.0. Sequencing libraries were generated using an Illumina HiSeqTM 2000 RNA Sample Preparation Kit (Illumina, San Diego, CA, USA) following the manufacturer’s recommendations and four index codes were added to attribute sequences to each sample. Differential expression analysis was performed using the NOIseq method (Sonia Tarazona 2100). Significant differentially expressed genes (DEGs) were defined as having a false discovery rate (FDR) < 0.05 and a log_2_ (the gene expression ratio of the Δ*aesR* mutant to WT) >1.2 or <−1.2. Gene Ontology (GO) enrichment analysis of the DEGs was implemented by the GOseq R package (version 1.3.1, Haibao Tang, Pasadena, CA, USA), in which the gene length bias was corrected. GO terms with corrected *p*-values less than or equal to 0.01 were considered to indicate significant enrichment of DEGs.

### 2.9. Electrophoretic Mobility Shift Assay (EMSA)

EMSA was performed using the method of Si et al. [4]. Increasing concentrations of AesR (0–120 nM) were mixed with 220 bp *P_aesR_* in 20 μL of gel shift buffer (10 mM Tris, 5 mM MgCl_2_, 50 mM KCl, 5% glycerol, 1 mM DTT and 5 µg/mL sspDNA, pH 7.4). A 221 bp fragment from the *aesR* coding region amplified with primers Control-F/Control-R instead of *P_aesR_* or BSA instead of AesR was added to binding reaction mixtures as negative controls. A 220 bp *P_aesRM_* (its start and stop sites were the same as those of 220 bp *P_aesR_*) was substituted for *P_aesR_* in EMSA. A 44 bp DNA sequence (Wild type, WT) and three mutated fragments (M1–M3) were first directly synthesized by Shanghai Biotechnology Co., Ltd. (Shanghai, China), each with disruption of one or two inverted repeats, and tested again in EMSAs with AesR. After 30 min of incubation at room temperature (RT), electrophoresis was performed with 8% to 15% native polyacrylamide gels in 0.5 × TBE buffer at 4 °C and 180 V for 60 to 90 min (depending on the size of the DNA fragments). The gels were stained with SYBR Gold dye (TransGen Biotech, Beijing, China). The DNA bands were visualized with UV light at 254 nm. For the testing of effector molecules, AesR was first incubated with various putative effectors at different concentrations for 30 min at RT, followed by addition of 40 nM DNA promoters, further 20 min incubation and electrophoresis as described above.

To test the effect of metal ions on the binding of AesR to the DNA promoters, EDTA was removed from the gel, the binding reaction buffer, and electrophoresis buffer. Simultaneously, EDTA (0.1 mM EDTA) and/or different concentrations of metal ions were added into the binding reaction.

For the determination of apparent K_d_ values, photographed were quantified using ImageQuant software (version 8.2) (GE Healthcare, Piscataway, NJ, USA), and the percentage of shifted DNA was calculated. These values were plotted against the AesR concentration in log10 scale, and a sigmoidal fit was performed using GraphPad Prism software (version 8.0, San Diego CA, USA), considering the error bars as well as 0 and 100% shifted DNA as asymptotes, the turning point of the curve was defined as the apparent K_d_ value. All determinations were performed in triplicate.

### 2.10. β-Galactosidase Assay

WT(pXMJ19), Δ*aesR*(pXMJ19), and Δ*aesR*(pXMJ19-*aesR*) cells transformed with the resulting *lacZY* fusion reporter vectors were grown in LB medium containing IPTG and corresponding antibiotics (Nal, Kan, and Chl) to OD_600_ of 0.6–0.7 for determination of β-galactosidase activity. For induced expression analysis, WT(*P_ncgl0018-ncgl0017_::lacZY*), WT(*P_aesR-ncgl0020_::lacZY*), Δ*aesR*(*P_ncgl0018-ncgl0017_::lacZY*), and Δ*aesR*(*P_aesR-ncgl0020_::lacZY*) strains were grown in triplicate in 100 mM Glc-containing MS medium until stationary phase and then harvested and transferred into 100 mM Glc-containing MS medium at a 1.0% inoculum in the presence of 0.5 mM copper chloride (CuCl_2_), 0.5 mM nickel sulfate (NiSO_4_), 1.0 mM zinc sulphate (ZnSO_4_), 0.15 mM cumene hydroperoxide (CHP), 0.25 mg/mL lincomycin (LIN), 5 ng/mL rifamycin (RIF), 360 μg/mL PEN, 1.0 μg/mL vancomycin (VAN), 1 mM 2, 5 dihydroxybenzoate (2, 5 HBA), 3 mM catechol (CAT), 1 μg/mL cefatenac (CEFA), 5 μg/mL ceftazidime (CEFT), and 1 µg/mL cephalosporins-cefuroxime (CEFU). Overnight cultures grown on 100 mM Glc-containing MS in the presence of various agents were used for analysis of β-galactosidase activity. β-galactosidase activities were measured with o-nitrophenyl-β-D-galactopyranoside (ONPG) as the substrate [17].

### 2.11. Quantitative Reverse Transcription-Polymerase Chain Reaction (qRT-PCR) Analysis

The qRT-PCR experiment was conducted according to the previous method [13]. Mid-log phase WT(pXMJ19), Δ*aesR*(pXMJ19) and Δ*aesR*(pXMJ19-*aesR*) strains cultivated in LB medium were used to isolate total RNA using the RNeasy Mini Kit (Qiagen, Hilden, Germany) along with the DNase I Kit (Sigma-Aldrich, Taufkirchen, Germany). For induced expression analysis, WT and Δ*aesR* strains were grown in triplicate in 100 mM Glc-containing MS until the stationary phase and subsequently harvested and transferred into 100 mM Glc-containing MS medium at a 1.0% inoculum in the presence of various agents. Overnight cultures grown on 100 mM Glc-containing MS in the presence of various agents were collected for RNA isolation. Purified RNA was reverse-transcribed with random 9-mer primers and MLV reverse transcriptase (TaKaRa, Dalian, China). Quantitative RT-PCR analysis (7500 Fast Real-Time PCR; Applied Biosystems, Foster City, CA) was performed as described previously [13]. The primers used were listed in Table S2. To obtain standardization of results, the relative abundance of 16S rRNA was used as the internal standard.

### 2.12. Statistical Analysis

The data are presented as means ± standard deviation (SD) (*n* = 3). Statistical analyses were performed using unpaired two-tailed Student’s *t*-test. Statistical analyses were performed using GraphPad Prism software (GraphPad Software, San Diego, CA, USA).

## 3. Results

### 3.1. AesR Was a MarR-Type Regulator Conserved in the Corynebacteriales

The 372 bp *C. glutamicum ncgl0019* gene, located at 19,705 to 20,076 bp, encodes encodes a 123-amino-acid, 13,870-Da predicted MarR homologue. Sequence analysis revealed that putative homologs of *aesR* gene were present in some species of the genus *Corynebacterium* as well as in some other bacteria, such as *C. glutamicum* SCgG1, *C. glutamicum* R, *C. halotolerans*, *C. efficiens*, *Dietzia psychralcaliphila*, and *Brevibacterium linens* (Figure S1A). Moreover, this corresponding genomic locus was highly conserved among *Corynebacterium* species, for example, in *C. halotolerans* and *C. efficiens*.

Figure S1B showed a sequence alignment of the putative AesR with characterized members of the MarR family. Despite sharing an average amino acid sequence similarity of less than 25% across the family, these proteins displayed pronounced structural homology. Helices α3 and α4 comprised the helix–turn–helix motif, whereof α4 served as the specific protein-DNA recognition helix. The ‘wing’ motif was formed by β2, W and β3. The ‘N/C-terminal domain’, formed by α-helical 1, α-helical 5 and α-helical 6 structural elements, was found to participate in dimerization, consistent with the typical dimeric architecture of MarR-like transcriptional regulators [18]. Furthermore, multiple sequence alignment of these proteins revealed that residues in α4 helix that made direct contacts with DNA bases had low conservation, indicating that DNA sequence recognition by AesR differed from those of other characterized MarR family proteins (Figure S2B). However, its physiological function and underlying regulatory mechanism remained elusive hitherto. Thus, these observations led us to investigate the role of this protein in *C. glutamicum*.

### 3.2. Influence of the aesR Deletion on Global Gene Expression

**Figure 1:**
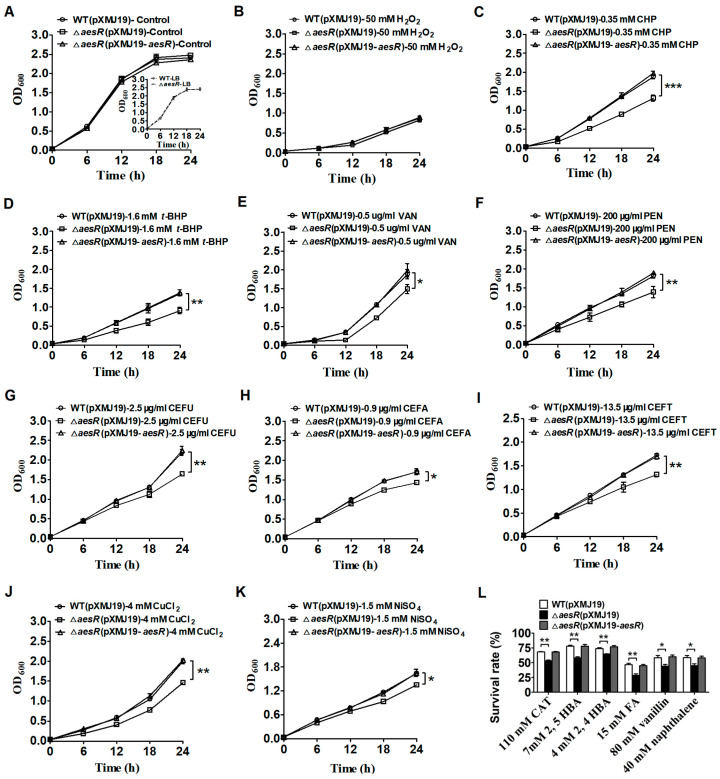
AesR was required for optimal growth under some stress. (**A**) Growth of WT(pXMJ19) strains (WT cells with empty pXMJ19 plasmids), the Δ*aesR*(pXMJ19) mutant (Δ*aesR* mutant expressing pXMJ19) and the complementary strains Δ*aesR*(pXMJ19-*aesR*) (the Δ*aesR* mutant expressing pXMJ19-*aesR*) in LB broth without stress was used as control. The inserted graph represents the growth curve of WT and Δ*aesR* cultivated in LB broth. (**B**–**K**) Growth of indicated strains in LB broth with different stress. (**L**) The indicated three strains grown to the stationary phase were exposed to aromatic compounds for 30 min at 30 °C, respectively. H_2_O_2_, hydrogen peroxide; CHP, cumene hydroperoxides; *t*-BHP, ter-butyl hydroperoxides; VAN, vancomycin; PEN, penicillin; CEFU, cefuroxime; CEFA, cefatenac; CEFT, ceftazidime; CAT, catechol; 2, 5 HBA, 2, 5 dihydroxybenzoate; 2, 4 HBA, 2, 4 dihydroxybenzoate; FA, ferulic acid. The viability of the cells was determined. Data show the averages of three independent experiments, and error bars indicate the SDs from three independent experiments. ***, *p* ≤ 0.001; **, *p* ≤ 0.01; *, *p* ≤ 0.05.

**Figure 2:**
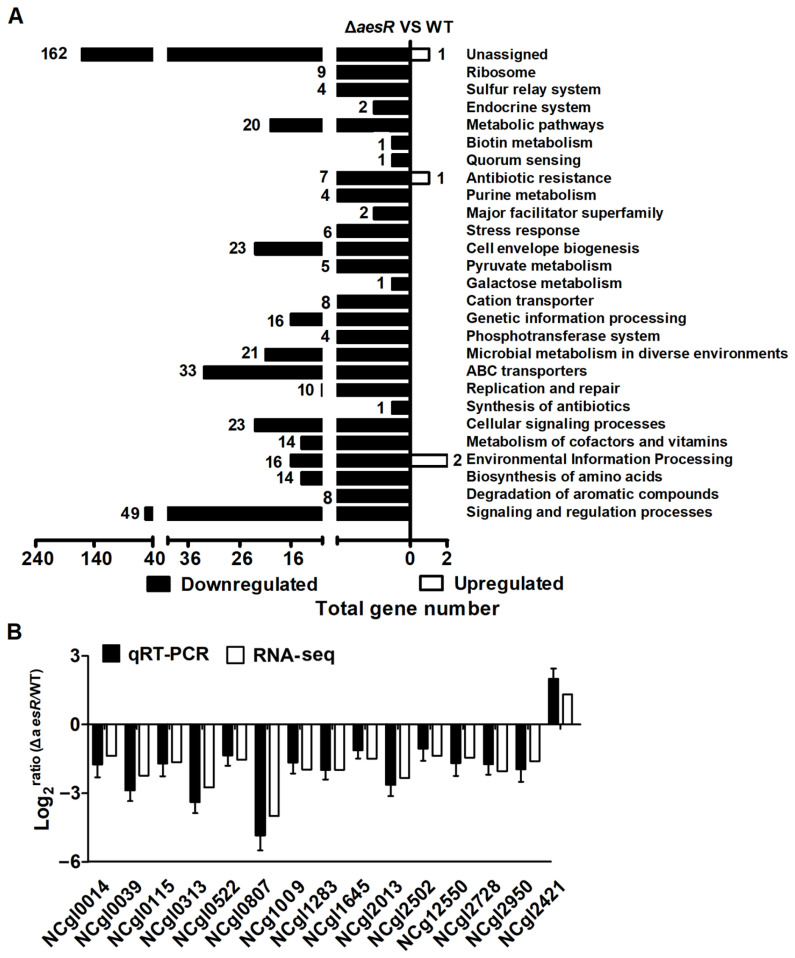
RNA-seq analysis of AesR regulated genes in *C. glutamicum.* (**A**) KEGG pathway analysis of differentially expressed genes (Δ*aesR* mutant vs. *C. glutamicum* RES167 parental strain (WT)). The black and white bars represent down- and up-regulated genes, respectively, and the numeric labels represent the number of genes related to that pathway. (**B**) Validation of RNA-seq data using qRT-PCR. Fifteen representative genes were evaluated for validation of the RNA-seq data using qRT-PCR.

To systematically identify the genes regulated by AesR and gain insights into its physiological function, we compared the transcriptome of the Δ*aesR* mutant with that of the *C. glutamicum* RES167 parent strain (WT) using RNA-seq-based transcriptomic analysis. The ∆*aesR* mutant, which was an in-frame deletion of the *aesR* gene in *C. glutamicum*, was constructed with the suicide vector pK18*mobsacB-*∆*aesR* via homologous recombination-based gene knockout (Figure S1C). For the transcriptome comparisons, total RNA was isolated from mid-exponential cultures of ∆*aesR* mutant and WT strain, which were cultured in LB medium. When cultivated under the condition, ∆*aesR* mutant exhibited a growth profile comparable to that of the wild-type strain, entering the stationary phase at a similar optical density (Figure 1A). A total of 468 DEGs (>2.3-fold alterations in the mRNA ratio with a *p*-value of <0.01) were identified Table S3). The functions of DEGs were identified by KEGG pathway analysis and 26 different pathways were found (Figure 2A). AesR from *C*. *glutamicum* appears to function as both a transcriptional repressor and activator, modulating the expression of genes participating in diverse cellular processes in this organism, such as metabolic pathways, ATP-binding cassette (ABC) transporters, cell envelope biogenesis, and signaling and regulation processes (Figure 2A). Importantly, about one-third of the 468 affected transcripts were those with unknown functions.

Among these genes, we found that the genes encoding proteins that were involved in antibiotic resistance, stress response and aromatic compound degradation, were downregulated in the Δ*aesR* mutant, supporting a hypothesis that AesR may be a non-negligible factor in giving *C. glutamicum* stress tolerance (Figure S2). To validate the transcriptomic profiles obtained from RNA-seq analysis, qRT-PCR was performed for fifteen randomly selected genes with altered mRNA levels in the Δ*aesR* mutant, namely, *ncgl0014*, *ncgl0039*, *ncgl0115*, *ncgl0313*, *ncgl0522*, *ncgl0807*, *ncgl1009*, *ncgl1283*, *ncgl1645*, *ncgl2013*, *ncgl2502*, *ncgl2550*, *ncgl2728* and *ncgl2950*, as examples of dowregulated genes; *ncgl2421* as example of upregulated gene. The log_2_-transformed mean values of qRT-PCR from three biological replicates for all genes were in good agreement with the log_2_-transformed fold changes in the RNA-seq data (Figure 2B), which confirmed the credibility of the RNA-seq data.

### 3.3. Response of ΔaesR Mutants to Stress-Causing Agents

To investigate the role of AesR in adverse stresses defense, the ability of *aesR*-deficient mutant to resist exposure to various agents was evaluated by analyzing the strain growth under a variety of stress conditions. As depicted in Figure 1C,D, the Δ*aesR*(pXMJ19) mutant (Δ*aesR* mutant expressing pXMJ19) showed a significant increase in sensitivity to OHPs-CHP and tert-butyl hydroperoxides (*t*-BHP) than WT(pXMJ19) strains (WT cells with empty pXMJ19 plasmids) and the complementary strains Δ*aesR*(pXMJ19-*aesR*) (the Δ*aesR* mutant expressing the wild-type *aesR* gene in an inducible promoter *P_tac_*-containing shuttle vector pXMJ19). However, there was no obvious difference in growth rate between the tested strains in the presence of inorganic oxidant hydrogen peroxide (H_2_O_2_) (Figure 1B). These results suggested that AesR is essential for resistance to OHPs stress in *C. glutamicum*.

In a next step, investigated whether deletion of *aesR* affects the antibacterial activity of various antibiotics against three *C. glutamicum* strains: WT(pXMJ19), Δ*aesR*(pXMJ19) and Δ*aesR*(pXMJ19-*aesR*). For all the tested antibiotics [glycopeptides (e.g., VAN), β-lactams (e.g., PEN, CEFU, CEFA, and CEFT), macrolides (e.g., ERY and LIN), quinolones (e.g., norfloxacin (NOR)), ansamitocins (e.g., RIF), and aminoglycosides (e.g., gentamicin (GEN))], VAN, PEN, CEFU, CEFA, CEFT, NOR, and GEN stress gave a marked growth inhibition for Δ*aesR*(pXMJ19) mutant than for WT(pXMJ19) and Δ*aesR*(pXMJ19-*aesR*) strains (Figure 1E–I and Figure S3A–E). Glycopeptides, β-lactams, quinolones, and aminoglycosides belonged to bactericidal antibiotics. These results led us to speculate AesR was involved in bactericidal antibiotics resistance in *C. glutamicum*.

The influence of heavy metal ions, including Cu^2+^, Ni^2+^, Mn^2+^, Zn^2+^, Cd^2+^, and Cr^6+^, on growth of the corresponding strains was also analyzed by determining turbidity of the cultures (OD_600_) in LB medium containing indicated concentration of heavy metal ions (Figure 1J–K and Figure S3F–H). Compared with the WT(pXMJ19), the Δ*aesR*(pXMJ19) mutant displayed significantly impaired growth under Cu^2+^ and Ni^2+^ stress, and this growth-impaired phenotype was restored by complementary strain Δ*aesR*(pXMJ19-*aesR*). Collectively, these data indicate that AesR participates in the resistance to Cu^2+^ and Ni^2+^ in *C. glutamicum*.

Finally, to investigate the physiological function of AesR in resistance to aromatic compounds, we assessed the survival of WT(pXMJ19), Δ*aesR*(pXMJ19) and Δ*aesR*(pXMJ19-*aesR*) strains in the presence of CAT, gentisate (also known as 2, 5 HBA), 2, 4 dihydroxybenzoate (2, 4 HBA), FA, vanillin, and naphthalene. As depicted in Figure 1L, disruption of *aesR* gene significantly reduced the tolerance of *C*. *glutamicum* to these conditions relative to WT(pXMJ19) strain. In contrast, the stress-hypersensitive phenotype of the Δ*aesR*(pXMJ19) mutant to these adverse stresses was largely restored by complementation with the *aseR* gene, resulting in a growth profile comparable to that of WT(pXMJ19) strain. These results indicate that AesR plays an essential role in defending *C. glutamicum* against aromatic compound stress.

### 3.4. AesR Negatively Regulated the Expression of the Divergently Oriented Operons ncgl0018-ncgl0017 and aesR-ncgl0020

We noticed that the start codon of *ncgl0020* gene overlapped the stop codon of *aesR* gene. A putative promoter-like element for *aesR* was identified that lied in 95 bp upstream of the start codon of *aesR* gene by the online promoter prediction tools TSSW, FPROM, and MEME suite. However, on further analysis of upstream of the *ncgl0020* gene, no other promoter sequence was predicted (Figure S4A). Based on these observations, we hypothesized that *aesR* and *ncgl0020* were likely transcribed as a polycistronic mRNA from the *aesR* promoter. Inspection of immediately upstream of *aesR* revealed two divergently transcribed open reading frames, *ncgl0018* and *ncgl0017*, coding a DsbA (thiol-disulfide oxidoreductase A) protein and a CytC (cytochrome C) biogenesis protein CcdA, respectively (Figure S4A). Thiol-disulfide oxidoreductase participates in disulfide bond reduction during the maturation of C-type cytochromes. It likely obtains reducing equivalents from CcdA, thereby facilitating the cleavage of disulfide bonds in apocytochrome C. This indicated that NCgl0018 and NCgl0017 might be involved in the pathway CytC assembly and protein modification. Moreover, the stop codon of *ncgl0018* and the initiation codon of *ncgl0017* are spaced by merely 4 bp, implying that these two genes may form an operon-like structure. To confirm the predicted *ncgl0018*-*ncgl0017* and *aesR*-*ncgl0020* operons, RT-PCR assay with cDNA as the template was performed using primers (Figure S5A). As shown in Figure S5B, products of the expected size were obtained, confirming the proposed operons.

**Figure 3:**
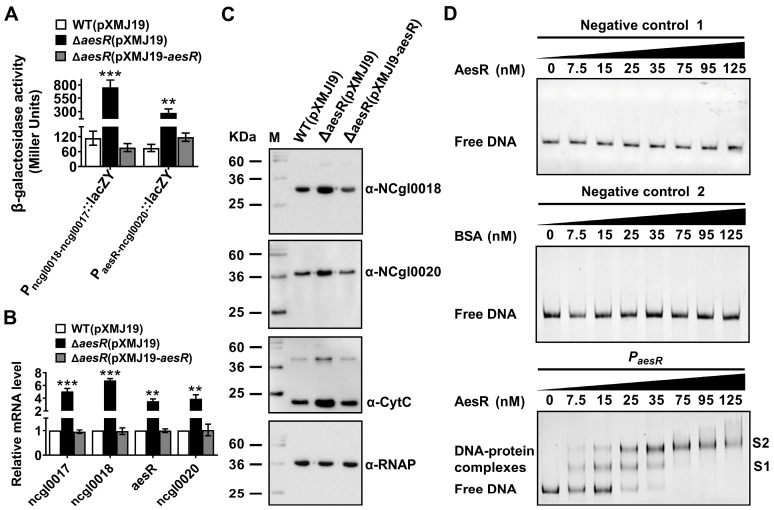
Negative regulation of the *ncgl0018-ncgl0017* and *aesR-ncgl0020* operons by AesR. (**A**) β-galactosidase analyses of the promoter activities of *ncgl0018-ncgl0017* and *aesR-ncgl0020* operons by using the transcriptional *P_ncgl0018-ncgl0017_::lacZY* and *P_aesR-ncgl0020_::lacZY* chromosomal fusion reporter expressed in WT(pXMJ19), Δ*aesR*(pXMJ19) and Δ*aesR*(pXMJ19-*aesR*) strains. Data show the averages of three independent experiments, and error bars indicate the SDs from three independent experiments.The asterisk indicated a significant correlation between the WT(pXMJ19) and ∆*aesR*(pXMJ19) strains at *** *p* ≤ 0.001 and ** *p* ≤ 0.01. (**B**) qRT-PCR analyses examining the transcription of *ncgl0017*, *ncgl0018*, *aesR*, and *ncgl0020* in WT(pXMJ19), Δ*aesR*(pXMJ19) and Δ*aesR*(pXMJ19-*aesR*) strains. The mRNA levels were presented relative to the value obtained from WT(pXMJ19) cells without stress treatment. Relative transcript levels of WT(pXMJ19) strains without stress treatment were set at a value of 1.0. Data show the averages of three independent experiments, and error bars indicate the SDs from three independent experiments. The asterisk indicated a significant correlation between the WT(pXMJ19) and ∆*aesR*(pXMJ19) strains at *** *p* ≤ 0.001 and ** *p* ≤ 0.01. (**C**) The protein levels of NCgl0018, NCgl0020, and cytC in WT(pXMJ19), Δ*aesR*(pXMJ19) and Δ*aesR*(pXMJ19-*aesR*) strains. Lysates from stationary phase bacteria were resolved by SDS-PAGE, and NCgl0018, NCgl0020, and CytC were detected by immunoblotting using specific anti-NCgl0018, anti-NCgl0020, anti-CytC antibodies. For the pellet fraction, RNA polymerase α (α-RNAP) was used as a loading control. Similar results were obtained in three independent experiments, and data shown were from one representative experiment done in triplicate. (**D**) AesR bound specifically to the intergenic region between *ncgl0018-ncgl0017* and *aesR-ncgl0020* operons. The interaction between AesR and the 220 bp promoter fragment in the intergenic region between *ncgl0018-ncgl0017* and *aesR-ncgl0020* operons (named *P_aesR_*, bottom panel). A 220 bp fragment amplified from the *aesR* coding region using the primers control F and control R instead of the 220 bp *aesR* promoter (upper panel) and an irrelevant protein BSA instead of AesR in the binding assays were used as negative controls (middle panel). AesR-*P_aesR_* DNA complexes were indicated as S1 and S2 at the right.

By RNA-seq-based newtrans and PROM-Prediction of bacterial promoter analysis, we identified two predicted overlapping and divergent promoter sequences within the 207 bp short intergenic region between the start codons of *aesR* and *ncgl0018* (Figure S4A). One of these elements was positioned upstream of *aesR* and likely serves as its native promoter. Neighboring *ncgl0018* was another deduced promoter elements [TGCCACAT (−10) and TTGCAT (−35)], which might be the promoter of the *ncgl0018-ncgl0017* operon. As mentioned above, RNA-seq analysis showed that the mRNA levels of *ncgl0017*, *ncgl0018* and *ncgl0020* were much higher in the Δ*aesR* mutant than those in the WT strain (Table S3). This demonstrated that not only *aesR* expression might be subjected to auto-regulation but also AesR repressed the transcription of the *ncgl0018*-*ncgl0017* operon. As shown in Figure 3A, the obviously increased levels of β-galactosidase activity (3.8~6.5-fold) were observed in the Δ*aesR*(pXMJ19) mutant compared with those in the WT(pXMJ19) and Δ*aesR*(pXMJ19-*aesR*) strains, suggesting that the originally proposed promoter regions were indeed functional as promoters of the *ncgl0018*-*ncgl0017* and *aesR*-*ncgl0020* operons and the two operons were negatively regulated by AesR.

We further performed qRT-PCR to evaluate the regulatory effect of AesR on the transcription of *ncgl0017*, *ncgl0018*, *aesR* and *ncgl0020* genes. Notably, to detect the expression of *aesR* in the Δ*aesR* mutant via qRT-PCR, an 89 bp *aesR* transcript (corresponding to nucleotides +1 to +89 relative to the translational start codon (ATG) of the *aesR* gene) was amplified from the residual *aesR* ORF in the Δ*aesR* mutant with the primers QaesR-F/QaesR-R (Figure S6). Figure 3B showed that the mRNA levels of *ncgl0017*, *ncgl0018*, *aesR*, and *ncgl0020* genes were obviously enhanced in strains Δ*aesR*(pXMJ19) compared with those in strains WT(pXMJ19) (3.9~6.6-fold). These results were further validated by monitoring the intracellular protein levels of NCgl0018 (anti-NCgl0018), NCgl0020 (anti-NCgl0020), or CytC (anti-cytC) in Western blot experiments with total cell lysates of the three *C. glutamicum* strains. In line with the data obtained for transcription control, the NCgl0018/NCgl0020/CytC levels were much higher in the Δ*aesR*(pXMJ19) mutant than those in the WT(pXMJ19) strain (Figure 3C and Figure S7). Collectively, these findings demonstrated that AesR negatively regulates the transcription of *ncgl0017*, *ncgl0018*, *ncgl0020*, and its structural gene.

### 3.5. AesR Bound Specifically to the Intergenic Region Between the ncgl0018-ncgl0017 and aesR-ncgl0020 Operons

To determine whether AesR controlled the transcription of the *ncgl0018-ncgl0017* and *aesR-ncgl0020* operons directly, we performed EMSAs using purified AesR and 220 bp DNA sequences (corresponding to intergenic region between *ncgl0018* and *aesR*; designated for *P_aesR_*). Figure 3D bottom panel shows AesR bound directly and specifically to *P_aesR_*. Further, inasmuch as the amount of the AesR increased, two sets of shifted protein-DNA complexes S1 and S2 were detected. The more rapidly migrating complex S1 was dominant at lower protein concentrations, but the predominant complex was the slower migrating S2 at higher protein concentrations. This indicated the presence of two binding sites in the region, which led to AesR binding to its target sequence as a low-molecular-weight dimer-DNA complex (S1) and a higher-molecular-weight double dimer-DNA complex (S2). The combination of AesR and a control DNA fragment amplified from the *aesR* ORF did not delay migration (Figure 3D, upper panel), and incubation of BSA instead of AesR with *P_aesR_* also did not result in DNA fragments being retarded (Figure 3D, middle panel), confirming the specificity of AesR for the intergenic DNA fragment *P_aesR_*. Since the putative binding site of AesR was located in the intergenic region of *ncgl0018* and *aesR*, which were orientated divergently (Figure S4), the *ncgl0018*-*ncgl0017* and the *aesR*-*ncgl0020* operons were directly regulated by AesR. This finding was in line with reports on over half of the identified MarR homologs, and may reflect a conserved characteristic of this regulator family [19].

### 3.6. Identification of the AesR-Binding Motif

**Figure 4:**
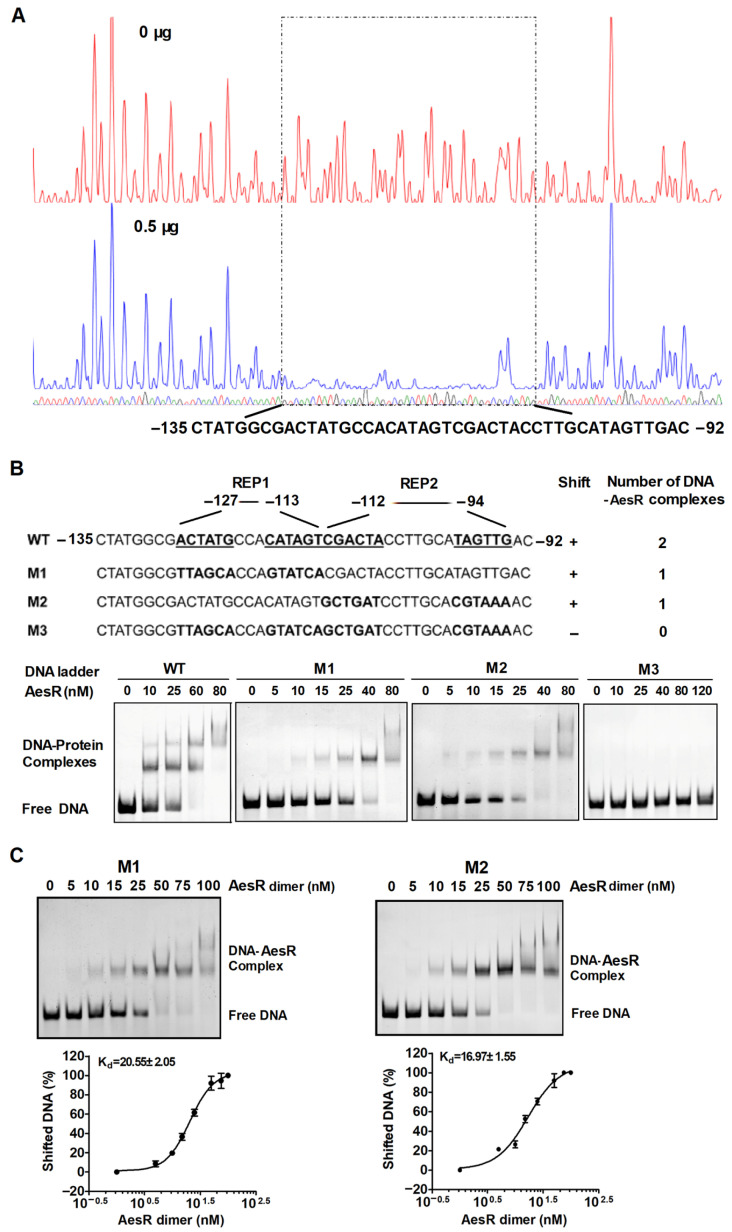
Identification of the AesR-binding site in the promoter regions of the *ncgl0018-ncgl0017* and *aesR-ncgl0020* operons. (**A**) Identification of the AesR-binding site within the promoter regions of the *ncgl0018-ncgl0017* and *aesR-ncgl0020* operons using the DNase I footprinting assay. (**B**) Mutational analysis of the AesR binding site within the promoter region of the *ncgl0018-ncgl0017* and *aesR-ncgl0020* operons. The mutations M1-M3 were introduced by PCR and shown below the wild-type sequence. The corresponding DNA fragments were analyzed by EMSAs with AesR. ‘+’ and ‘−’ signs indicated whether the fragment was shifted by AesR. (**C**) Determination of the apparent K_d_ values of AesR for M1 and M2. M1 and M2 were incubated with increasing AesR concentrations, resolved on an 8% native polyacrylamide gel, and stained with GelRed^TM^. At least three independent gels were performed for each binding site. The bands were quantified using ImageQuant software, and the percentage of shifted DNA was calculated from three independent gels. These values were plotted against the AesR concentration in log_10_ scale, and a sigmoidal fit was performed. The turning point of the curve was defined as the apparent K_d_ value.

To locate the precise binding site of AesR in the intergenic region between the *ncgl0018-ncgl0017* and *aesR-ncgl0020* operons, DNase I footprint analysis was performed (Figure 4A). A specific 44 bp DNA sequence, CTATGGCGACTATGCCACATAGTCGACTACCTTGCATAGTTGAC, was protected by AesR (Figure 4A). To prove that AesR bound the 44 bp DNA sequence, the 220 bp promoter DNA fragments containing the mutations in DNA sequence-protecting AesR (*P_aesRM_*) were chemically synthesized, and directly used for EMSA assays. As shown in Figure S8A, *P_aesRM_* abolished the formation of DNA-protein complexes in the EMSA assay. Consistently, the mutations in promoter DNA sequence-protecting AesR led to the high β-galactosidase activities in the WT(pXMJ19)(*P_ncgl0018-ncgl0017M_::lacZY*)/WT(pXMJ19)(*P_aesR-ncgl0020M_*::*lacZY*) and Δ*aesR*(pXMJ19-*aesR*)(*P_ncgl0018-ncgl0017M_::lacZY*)/Δ*aesR*(pXMJ19-*aesR*)(*P_aesR-ncgl0020M_*::*lacZY*) strains, which was comparable to that in the Δ*aesR*(pXMJ19)(*P_ncgl0018-ncgl0017M_::lacZY*)/Δ*aesR*(pXMJ19)(*P_aesR-ncgl0020M_*::*lacZY*) mutant (Figure S8B). Thus, results verified that AesR directly regulates both the *ncgl0018*-*ncgl0017* and *aesR*-*ncgl0020* operons in *C. glutamicum*. These results further indicated that AesR binding site was located within the corresponding sequence.

The binding sites (operators) of MarR-type regulators were featured by palindrome sequences [19]. Careful examination of the protected DNA region extending from −135 to −92 bp upstream of the ATG start codon of *aesR* ORF revealed two side-by-side 6 bp inverted repeats-REP1 (ACTATGCCACATAGT) and REP2 (CGACTACCTTGCATAGTTG)-which might be related to the AesR operator. Thus, the 44 bp DNA fragment was termed as operator *aesRO*. The *aesR* operator site is fully contained within the spacer region between the −35 and −10 elements, the two critical promoter motifs recognized by RNA polymerase. REP1 and REP2 sequences overlapped part or all of the putative promoter signatures (for the *ncgl0018-ncgl0017* or *aesR-ncgl0020* operon) (Figure S5A). The relevance of two side-by-side inverted repeats with the binding of AesR was analyzed by mutational analysis. The EMSA results showed that DNA fragments containing the inverted repeats REP1 and REP2 (WT fragments) formed two AesR-DNA complexes (Figure 4B). When REP1 or REP2 was truncated, as represented by M1 and M2 fragments, only one shifted species was observed in each case, demonstrating that both fragments were *bona fide* AesR-binding sites and AesR bound to M1 or M2 fragment (Figure 4B). However, disruption of both side-by-side inverted repeats (as in the case of M3) resulted in the failure of the AesR binding. The result was in perfect agreement with that for the two AesR-DNA complexes found in the EMSA assay (Figure 3D, right panel). From these experiments, we also calculated dissociation constants (K_d_) of 20.55 ± 2.05 nM and 16.97 ± 1.55 nM for the M1 and M2, respectively (Figure 4C). These apparent K_d_ values were within the range found for other MarR family members [20]. These data also showed that the binding capacity of AesR for the perfect palindrome REP1 (ACTATGCCACATAGT) was higher than that for the imperfect palindrome REP2 (CGACTACCTTGCATAGTTG), suggesting that AesR bound with different affinities to both sites. Considering the existence of native AesR as homodimer (Figure S9) and the presence of side-by-side palindromes (Figure 4B), we speculated that two complexes S1 and S2 corresponded to binding of one homodimer and a pair of homodimers, respectively. Together, these results indicated that AesR could not only bind to any single inverted repeat (REP1 or REP2), but also the whole *aesRO* fragment.

Using the MEME program, we searched the upstream regions of *aesR* homologs in *C. halotolerans*, *C. efficiens*, *D. psychralcaliphila* and *B. linens* for potential AesR-binding motifs. A conserved 34 bp sequence was identified, harboring two 6 bp inverted repeats that resembled the binding site characterized upstream of the *aesR* gene in *C. glutamicum*.

Based on the sequence alignment presented in Figure S10, a consensus binding sequence for AesR was identified, which included two conserved perfect palindromic repeats composed of 6 bp half-sites [5΄-ACTATG-N_3_-CATAGTCGACTA-N_7_-TAGTCG-3΄]. The consensus sequence was distinct from those found to interact with other MarR-type regulators, such as *C. glutamicum* RosR [20], *Neisseria adhesin* FarR (fatty acid resistance regulator) [21], *Sinorhizobium meliloti* OhrR [22], and *E. coli* HpaR [23]. In addition, to identify further AesR target genes, the binding motif 5′-ACTATG-N_3_-CATAGTCGACTA-N_7_-TAGTCG-3′ was used for a genome-wide in silico search using the ERGO^TM^ bioinformatics suite (Integrated Genomics, Chicago, Illinois, USA) (allowing two mismatches, no deletions and no insertions) for similar sequences within putative or identified promoter regions. Unfortunately, no hits were found, indicating that genes with altered mRNA levels in the RNA-seq analysis were not direct target genes of AesR. Thus, AesR had only four regulons *ncgl0017*, *ncgl0018*, *ncgl0020*, and its own.

### 3.7. Cu^2+^/Ni^2+^/Zn^2+^ Triggered the Dissociation of AesR from DNA

**Figure 5:**
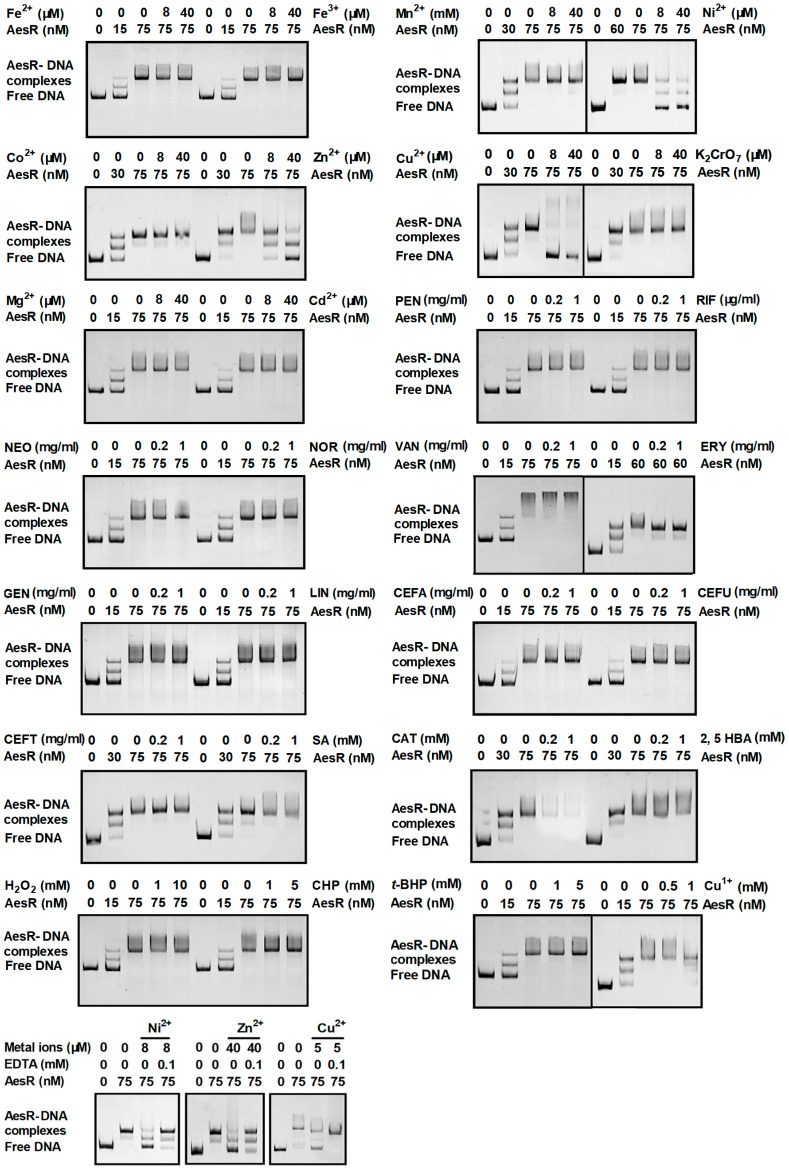
AesR-DNA complex was disrupted by copper (II), Zinc (II) and nickel (II). AesR was prepared in different concentrations, and aliquots were taken for EMSAs (control). Then, various ligands in different concentrations were added to the binding reaction mixture, and aliquots were taken for EMSA. RIF, rifamycin. NOR, norfloxacin. NEO, neomycin. ERY, erythromycin. GEN, gentamicin. LIN, lincomycin. SA, salicylic acid.

MarR family members typically regulated transcription through ligand-mediated attenuation of DNA binding and exhibited promiscuity in ligand binding [19]. Thus, we wondered whether reagents associated with physiological phenotype could induce AesR’s dissociation from its cognitive operator *aesRO*. We further explored whether AesR could respond to other potential ligands. We applied EMSAs to 40 nM DNA in the presence of AesR with and without the addition of multiple agents, including heavy metals, antibiotics, aromatic compounds, and oxidants. As shown in Figure 5, 8 µM Cu^2+^ significantly promoted association AesR with its cognate DNA; the promoter–DNA complex formation was slightly affected at 8 µM Zn^2+^ and 1 mM Cu^1+^; 8 µM Ni^2+^ or 40 µM Zn^2+^ was able to moderately interfere with the promoter–DNA complex interaction. It was to be noted that 40 µM Ni^2+^ did not neutralize the interaction between the operator/promoter and AesR. Furthermore, none of Fe^2+^, Fe^3+^, Mn^2+^, Co^2+^, Mg^2+^, and Cu^1+^ (high concentration, 40 µM or 0.5 mM) were found to cause AesR-DNA dissociation. We therefore ruled out the possibility that antibiotic-damaged Fe-S clusters release free iron, acting as a physiological signaling molecule for AesR. EMSA was then used to test antibiotics and aromatic compounds. Unexpectedly, despite the facts that PEN and salicylic acid (SA) were ligands of many characterized MarR family members [19], addition of up to millimolar concentrations did not affect stability of the AesR-DNA complexes. Moreover, we found that other antibiotics, including RIF, NEO (neomycin), NOR, VAN, ERY, GEN, LIN, and β-lactams (CEFT, CEFU and CEFTA), and other aromatic compounds, including CAT and 2, 5-HBA, did not cause the association of AesR from the promoters. Notably, in the presence of H_2_O_2_, CHP, or *t*-BHP, 15 µM Cu^1+^ was able to efficiently dissociate AesR from promoter DNA, whereas only 0.5 mM Cu^1+^, 10 mM H_2_O_2_, 5 mM CHP, or 5 mM *t*-BHP, did not attenuate AesR’s DNA-binding ability (Figure S11). This result was consistent with previous studies showing that oxidants oxidized cuprous ions to cupric species via the Fenton-like reaction, similar to the conversion of iron (II) to iron (III), to cause protein-DNA dissociation [24,25]. To confirm that Cu^2+^, Ni^2+^ and Zn^2+^ triggered AesR dissociation, we added 0.1 mM of metal-chelator ethylenediaminetetraacetic acid (EDTA) to reaction mixture of EMSAs in vitro. The result showed that the addition of Cu^2+^, Ni^2+^ or Zn^2+^ in the presence of 0.1 mM EDTA had no or extremely minor effect on the operator/promoter-AesR interactions as EDTA chelated the divalent ions and left AesR free to interact with the operator/promoter molecules to form the complexes. Taken together, these results revealed that Cu^2+^/Ni^2+^/Zn^2+^ attenuated AesR’s DNA-binding ability, Ni^2+^ and Zn^2+^ of which were weak disrupters (i.e., they caused a moderate shift of DNA towards less retarded forms at concentrations as high as 40 µM), and Cu^2+^ was a strong disrupter (i.e., it completely interfered with binding at concentrations as low as 8 µM).

### 3.8. Expression of the ncgl0018-ncgl0017 and aesR-ncgl0020 Operons Was Induced by Metal Ions Cu^2+^/Ni^2+^/Zn^2+^, OHPs, Bactericidal Antibiotics, and Aromatic Compounds via AesR

**Figure 6:**
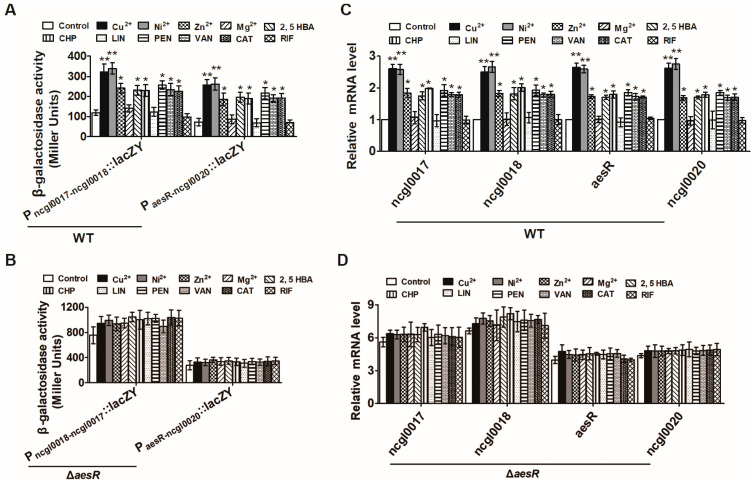
Expression of the *ncgl0018-ncgl0017* and *aesR-ncgl0020* operons was induced by various stress in an AesR-dependent manner. (**A**,**B**) β-galactosidase analysis of the promoter activities of the *ncgl0018-ncgl0017* and *aesR-ncgl0020* operons by using the transcriptional *P_ncgl0018-ncgl0017_::lacZY* and *P_aesR-ncgl0020_::lacZY* chromosomal fusion reporter expressed in WT and Δ*aesR* strains exposed to less than subinhibitory concentrations of various stress. (**C**,**D**) qRT-PCR assay was performed to analyze the expression of the *ncgl0017*, *ncgl0018*, *aesR*, and *ncgl0020* in WT and Δ*aesR* strains exposed to less than subinhibitory concentrations of various stress. The mRNA levels are presented relative to the value obtained from WT cells without treatment. Relative transcript levels of WT strains without stress treatment were set at a value of 1.0. Data show the averages of three independent experiments, and error bars indicate the SDs from three independent experiments. Statistical significance was calculated with respect to agent-untreated cells. **, *p* ≤ 0.01; *, *p* ≤ 0.05.

To prove whether Cu^2+^/Ni^2+^/Zn^2+^ was the cognitive signal for AesR derepression inside *C. glutamicum* and examine whether the expression of the *ncgl0018-ncgl0017* and *aesR-ncgl0020* operons responded to other xenobiotics at the transcriptional level, qRT-PCR profiling was performed, and the LacZY activities of the chromosomal promoter fusion reporter strain were determined with and without xenobiotics. For simplicity, we used less than subinhibitory concentrations of Cu^2+^, Ni^2+^, Zn^2+^, Mg^2+^, 2, 5 HBA, CHP, LIN, PEN, VAN, CAT, RIF, and β-lactams (CEFT, CEFU and CEFTA) as inducers in the following experiments. As shown in Figure 6A, the addition of Cu^2+^, Ni^2+^, and Zn^2+^ led to over 1.6-fold increase of β-galactosidase activity in WT(*P_aesR-ncgl0020_::lacZY*) and WT(*P_ncgl0018-ncgl0017_::lacZY*) strains compared to untreated cells or cells treated with Mg^2+^, LIN, or ERY_._ Similarly, 2, 5 HBA, CHP, PEN, VAN, CAT, and β-lactams (CEFT, CEFU, CEFTA) were also found to stimulate over 1.5 times greater β-galactosidase activities in WT(*P_aesR-ncgl0020_::lacZY*) and WT(*P_ncgl0018-ncgl0017_::lacZY*) strains compared to untreated samples (Figure 6A and Figure S12A). The elevated β-galactosidase activity observed under induction (Figure 6A and Supplementary Figure S12A) was consistent with the increased mRNA levels in the WT cells induced by Cu^2+^, Ni^2+^, Zn^2+^, 2, 5 HBA, CHP, PEN, VAN, CAT, and β-lactams (CEFT, CEFU, CEFTA) (Figure 6A and Figure S12A), demonstrating that Cu^2+^, Ni^2+^, Zn^2+^, 2, 5 HBA, CHP, PEN, VAN, CAT, and β-lactams (CEFT, CEFU, CEFTA) induced the expression of the *aesR-ncgl0020* and *ncgl0018-ncgl0017* operons in this pathway. Finally, the Δ*aesR*(*P_aesR-ncgl0020_::lacZY*) and Δ*aesR*(*P_ncgl0018-ncgl0017_::lacZY*) mutants were used as controls, which exhibited only a negligible increase of β-galactosidase activities and of mRNA levels after Cu^2+^, Ni^2+^, Zn^2+^, 2, 5 HBA, CHP, PEN, VAN, CAT, and β-lactams (CEFT, CEFU, CEFTA) treatment (Figure 6B,D and Figure S12B,D). Interestingly, *ncgl0018* was induced by different concentrations of CHP stress in an oxidative stress-sensing OasR (organic peroxide- and antibiotic-sensing regulator)-dependent manner, while *ncgl0018* transcript in Δ*aesR* mutant in the presence of 0.15 mM CHP was indistinguishable from that in the absence of CHP, indicating that OasR might be effective at high concentrations of CHP [26].

Interestingly, the xenobiotics-mediated AesR derepression in vivo was inconsistent with the results of EMSAs in vitro, which showed that the addition of 2, 5 HBA, CHP, PEN, VAN, CAT, and β-lactams (CEFT, CEFU, CEFTA) did not cause the dissociation of AesR from the promoters (Figure 5, Figure 6 and Figure S12). A report found that bactericidal antibiotics (e.g., NOR and AMP), OHPs (e.g., CHP and *t*-BHP) and aromatic compounds (e.g., SA) could cause the impairment of cytoplasmic membrane-bound copper proteins and lead to an elevated intracellular copper level in *E. coli* cells [27]. In combination with the facts that the *ctaD*, *ctaC*, and *ctaE* genes encoding subunit I, II, and III of cytochrome aa3 oxidase on *C. glutamicum* cell envelope belonged to the heme-copper proteins, and there were many copper/zinc ion-binding member proteins, such as Cg3242 [28]. Thus, we speculated that xenobiotics-OHPs, bactericidal antibiotics and aromatic compounds triggered an elevated intracellular Cu^2+^/Ni^2+^/Zn^2+^ level and subsequently Cu^2+^/Ni^2+^/Zn^2+^ induced AesR-DNA dissociation. For verifying the possibility, PEN was chosen to carry out the following experiments.

### 3.9. Clinically Important Antibiotic PEN Triggered the Increase of Intracellular Cu^2+^/Ni^2+^/Zn^2+^

**Figure 7:**
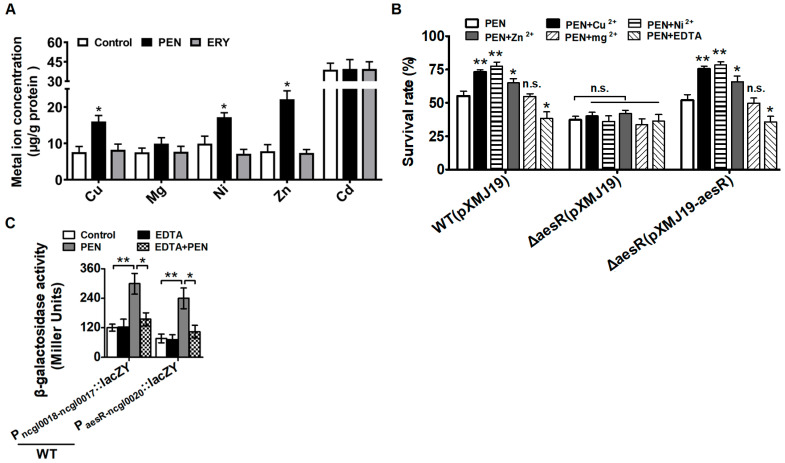
PEN triggered intracellular copper, nickel, and zinc elevation. (**A**) Stationary phase *C. glutamicum* strains were exposed to 2.5 mg/mL PEN and 320 μg/mL ERY for 30 min in PBS. Various ions in bacterial cells were measured by inductively coupled plasmon resonance atomic absorption spectrometry (ICP-MS). Data show the averages of three independent experiments, and error bars indicate the SDs from three independent experiments. Statistical significance was calculated with respect to agent-untreated cells. *, *p* ≤ 0.05. (**B**) Alleviation of the sensitivity of *C. glutamicum* strains to PEN by exogenous ions required AesR. Relevant stationary-phase bacterial strains were exposed to 2.5 mg/mL PEN in PBS with or without exogenously provided ions (1 μM) or EDTA (1 mM), and the viability of the cells was determined. Statistical significance was calculated with respect to agents-untreated cells. Data show the averages of three independent experiments, and error bars indicate the SDs from three independent experiments. Statistical significance was calculated with respect to PEN-treated cells. **, *p* ≤ 0.01; *, *p* ≤ 0.05; n.s., not significant. (**C**) β-galactosidase analysis of the promoter activities of the *ncgl0018-ncgl0017* and *aesR-ncgl0020* operons by using the transcriptional *P_ncgl0018-ncgl0017_::lacZY* and *P_aesR-ncgl0020_::lacZY* chromosomal fusion reporter expressed in WT strain upon the addition of PEN with and without EDTA (1 mM). Data show the averages of three independent experiments, and error bars indicate the SDs from three independent experiments. The asterisk indicates a significant correlation between the PEN-treated and -untreated strains or the PEN-treated and EDTA + PEN-treated strains at ** *p* ≤ 0.01, * *p* ≤ 0.05.

We first determined the total metal content in bacterial cells treated with PEN by inductively coupled plasmon resonance atomic absorption spectrometry (ICP-MS). Our results showed that PEN treatment significantly increased intracellular Cu/Ni/Zn levels in *C. glutamicum* WT strains, whereas the addition of EYR did not generate a noticeable increase (Figure 7A). Exogenous Cu^2+^/Ni^2+^/Zn^2+^(1μM) increased the survival rate of the WT(pXMJ19) and the complemented strain Δ*aesR*(pXMJ19-*aesR*) under PEN challenge, the protective effect of exogenous Cu^2+^/Ni^2+^/Zn^2+^ was largely abolished in the Δ*aesR*(pXMJ19) mutant (Figure 7B). In contrast, when Mg^2+^ was added to the WT(pXMJ19) strains expose to PEN, we observed no detectable increase in survival rate. Further, the survival rate of the WT(pXMJ19) strains under EDTA and PEN treatment was markedly lower in comparison with that under only PEN treatment. This indicated that Cu^2+^/Ni^2+^/Zn^2+^ could enhance AesR-mediated bacterial drug resistance. To further confirm that Cu^2+^/Ni^2+^/Zn^2+^ was the real signal for PEN-triggered AesR derepression, we added 1 mM EDTA to the PEN-treated bacterial cells, which effectively diminished PEN-mediated AesR derepression (Figure 7C), thus verifying that PEN indirectly derepresses AesR via Cu^2+^/Ni^2+^/Zn^2+^ species within *C. glutamicum*. We will further identify the source of Cu^2+^/Ni^2+^/Zn^2+^ under xenobiotic-induced stress. Together, we revealed that Cu^2+^/Ni^2+^/Zn^2+^ could dissociate AesR-*aesR*O complex, thereby releasing AesR from the complex and derepressing the transcription of the operons in *C. glutamicum.* This indicated that Cu^2+^/Ni^2+^/Zn^2+^ served as a natural inducer for AesR and mediated the PEN-induced AesR derepression.

## 4. Discussion

Although the function of MarR resistance to environmental stress in *C. glutamicum* is well characterized, its regulatory mode and intrinsic inducer for *C. glutamicum* MarR in modulating bacterial resistance to diverse, structurally unrelated antibiotics, toxic chemicals, or both remained largely undiscovered. Here, we described a novel type of transcriptional regulator AesR, which acted as an environmentally responsive repressor to control the *ncgl0018*-*ncgl0017* operon responsible for cytochrome C synthesis, *aesR*-*ncgl0020* operon, and *ncgl2421* gene, and participated in the activation of many target genes most likely associated with degradation of aromatic compounds, stress response, antibiotic resistance, and cell envelope biogenesis in *C. glutamicum*.

Combined with the RNA-seq data and survival rate, we speculated that the increased sensitivity of Δ*aesR* mutant to aromatic compounds was related to down-regulated expression of genes involved in degradation of aromatic compounds, such as *c23o* (*ncgl2007*), *genR* (*ncgl2921*) and *vanA* (*ncgl2300*). C23O belongs to extradiol-type dioxygenases, which cleave aromatic C-C bond atmetaposition of dihydroxylated aromatic substrates and catalyzed the conversion of catechol to 2-hydroxymuconic semialdehyde [29]. The IclR-family regulator GenR has been reported to activate the expression of gentisate 1,2-dioxygenase (NCgl2920), maleylpyruvate isomerase (NCgl2918), and fumarylpyruvate hydrolase (NCgl2919), which were related with 2, 5 HBA/3-hydroxybenzoate catabolism [11]. Deletion of *genR* resulted in the loss of *C. glutamicum*’s ability to grow on 2, 5 HBA and 3-hydroxybenzoate. 2, 5 HBA and substituted 2, 5 HBA were key intermediates in aerobic degradation of numerous aromatic compounds, such as 3-hydroxybenzoate, xylenol, and naphthalene [11]. Shen et al. revealed that *C. glutamicum* degraded FA and vanillin to vanillic acid [10]. VanA could metabolize vanillic acid into protocatechuic acid, which was further degraded by the β-ketoadipic acid pathway. The above results indicate that AesR plays a significant role in regulating the degradation of aromatic compounds derived from lignocellulose by *Corynebacterium glutamicum*.

Deletion of the *aesR* gene resulted in a strongly increased sensitivity of *C. glutamicum* towards OHPs, bactericidal antibiotics, Cu^2+^, Ni^2+^, and aromatic compounds (Figure 1). In combination with the RNA-seq-based transcriptomics analysis (Table S3), we found that the enhanced sensitivity of the Δ*aesR* mutant to OHPs and bactericidal antibiotics (VAN, β-lactam antibiotics, NOR, and GEN) was consistent with the down-regulated expression of many of β-lactam antibiotic metabolism-involving proteins [NCgl1027, NCgl1321, NCgl1586, NCgl2422, and MalR (NCgl2886)] [30], OHPs-detoxifying peroxidases, such as Ohr (NCgl0023) [31], PrxQ (NCgl2403) [32], and MPx (NCgl2502) [33], the peroxidase activity-supporting proteins Trx1 (NCgl2985) [31,32,33] and TrxR (NCgl2984) [31,32,33], and cell envelope biogenesis proteins (NCgl0350, NCgl0352, and NCgl2046, etc.). Interestingly, *ncgl0018* exhibited up-regulating expression in the Δ*aesR* mutant and *ncgl0018*-disrupting strain was found to be sensitive to 5.5 mM CHP [26], while deletion of *aesR* overtly affected the ability of *C. glutamicum* to grow in LB medium in the presence of 0.35 mM CHP, which indicated that NCgl0018 expression did not provided a CHP resistance phenotype and low levels of other antioxidant proteins were not sufficient to maintain the resistance in Δ*aesR* mutant. This might be because NCgl0018 reduced disulfide bond in antioxidant protein coupled to the reducing mycoredoxin-1 (Mrx1) system (Mrx1/mycothione reductase (Mtr)/mycothiol (MSH)/NADPH), but genes related to the reducing Mrx1 system showed no changed expression in Δ*aesR* mutant; thus, the influence of *ncgl0018* overproduction alone on the oxidative damage should be marginal in the Δ*aesR* mutant strain. Previous studies have shown that many bactericidal antibiotics exerted their bactericidal effects by generating oxidative stress through hydroxyl radical (·OH) and OHPs formation [34]. Moreover, relies on multiple antioxidant defense systems, such as a thick cell wall rich in lipoarabinomannan, cyclopropanated mycolic acid, and phenolic glycolipid I (PGL-1) [35], as well as protective enzymes [3,4]. Thus, we speculated that excessive toxic substances were imported into the cells, and excessive toxic substances and reactive oxidative species-·OH and OHPs produced by toxic substances could not be eliminated, which caused an increased sensitivity of deletion mutant (Δ*aesR*) to OHPs and bactericidal antibiotics. RNA-seq-based transcriptomic analysis also showed the decreased expression of genes encoding proteins associated with copper export (*ncgl0465* and *ncgl2859*), genes encoding Cu-containing proteins (*ncgl2112*, *ncgl2115*, *ncgl2437*, and *ncgl2875*), *ncgl2865* coding for hypothetical multicopper oxidase, and *ncgl0221* encoding Ni of exported protein in the Δ*aesR* mutant. This indicated that reduced Cu^2+^/Ni^2+^ tolerance observed following disruption of *aesR* was related to increased ions accumulation. However, there were reduced expression of genes encoding zinc export proteins (*ncgl2052* and *ncgl2685*), genes encoding Zn-containing proteins (*ncgl0313* and *ncgl1027*), and *ncgl2200* gene encoding zinc import proteins in the Δ*aesR* mutant. Moreover, *ncgl0020* encoding Zn-dependent protease with chaperone function was up-regulated in the Δ*aesR* mutant. Thus, we speculated that the Δ*aesR* mutant did not accumulate too much zinc under zinc treatment.

Note that the purified AesR proteins with an N-terminal His_6_ tag or His_6_-SUMO tag were extremely unstable during the purification process or during storage at either 4 or −80 °C, which formed more aggregates and precipitates than His_6_- or His_6_-SUMO-free protein. Consequently, the tag was removed for experiments requiring prolonged protein stability to ensure reproducible results, including EMSA, measurement of apparent dissociation constants (K_d_), or analysis of the reversibility of DNA-binding inhibition by chemical agents. (Figure S9A,B). The native AesR proteins were eluted at a volume corresponding to ~29.3 kD by size exclusion chromatography (Figure S9C,D), suggesting homodimeric formation.

AesR was highly conserved in diverse *Corynebacterium* as well as in some other bacteria, such as *C. glutamicum* SCgG1, *C. glutamicum* R, *C. halotolerans*, *C. efficiens*, *Dietzia psychralcaliphila*, and *Brevibacterium linens* (Figure S1A). Moreover, the *aesR*-like regulatory genes were transcribed in the opposite direction relative to their putative target genes, and the *aesRO*-like motifs were located at the intergenic regions of the *aesR*-like and immediate upstream structural gene in *C. glutamicum* SCgG1, *C. glutamicum* B253, and *C. glutamicum* R, (Figures S1A and S10). The *aesRO*-like motifs and/or similar genetic organizations suggested that Cu^2+^/Ni^2+^/Zn^2+^ signal-mediated stress resistance may serve as a widespread mechanism for relieving repression by such MarR transcription factors in *Corynebacterium*.

## 5. Conclusions

In this study, we identify and characterize AesR, a novel MarR-type transcriptional regulator conserved across the *Corynebacteriales* order. Through integrated transcriptomic and phenotypic analyses, we demonstrate that AesR functions as a dual transcriptional regulator, governing genes involved in metabolism, transport, envelope biogenesis, stress defense, and antibiotic resistance. Deletion of *aesR* markedly sensitizes *C. glutamicum* to organic hydroperoxides, bactericidal antibiotics (e.g., vancomycin, β-lactams, quinolones, aminoglycosides), heavy metals (Cu^2+^, Ni^2+^), and aromatic compounds—defects fully rescued by genetic complementation.

Mechanistically, AesR directly represses two divergently oriented operons by binding a conserved 44 bp operator containing adjacent inverted repeats, with sequential homodimer binding at two sites. Critically, we identify Cu^2+^, Ni^2+^, and Zn^2+^ as physiological inducers that trigger AesR–DNA dissociation, among which Cu^2+^ is most potent. While organic hydroperoxides, antibiotics (e.g., penicillin), and aromatic compounds induce AesR derepression in vivo, they fail to disrupt binding in vitro. Instead, we reveal that penicillin elevates intracellular Cu^2+^/Ni^2+^/Zn^2+^ levels, establishing these metal ions as the bona fide signaling molecules that couple antibiotic stress to AesR regulation.

**Figure 8:**
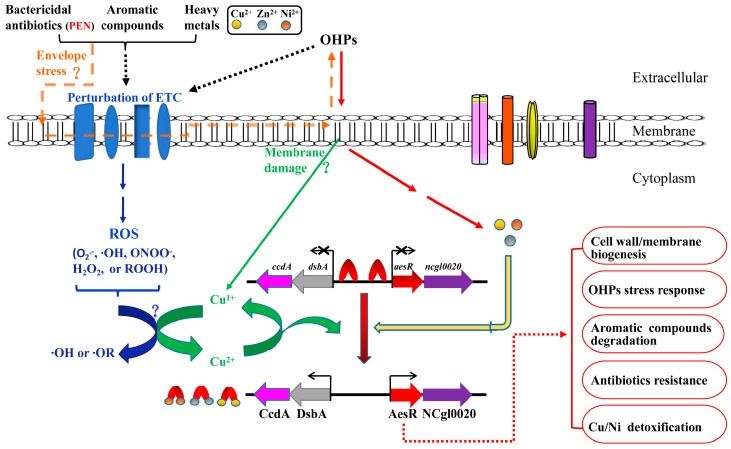
Proposed mechanistic model for stress-triggered metal ion signals Cu^2+^/Ni^2+^/Zn^2+^ and AesR-mediated stress resistance in C. glutamicum. Clinically important antibiotic PEN and OHPs could stimulate the production of higher level of Cu^2+^/Zn^2+^/Ni^2+^ within the *C*. *glutamicum* cytosol. The binding of Cu^2+^/Ni^2+^/Zn^2+^ to AesR triggered the dissociation of AesR-DNA complex, thereby disinhibiting transcription of the *aesR-ncgl0020* operon and of the adjacent but divergently oriented *ncgl0018-ncgl0017.* The translation products AesR then activated the expression of many genes involved in cell wall/cell membrane biosynthesis, antibiotic resistance, OHP stress response, aromatic compound degradation, and copper/nickel detoxification (e.g., export, converting Cu^1+^ to the less toxic Cu^2+^, and synthesis of Cu/Ni-containing proteins) indirectly, which in turn contributed to bacterial antibiotic or environment-derived stress resistance. The red dotted line indicated indirect regulation. The membrane-associated copper oxidation and liberation process drew on the result of Hao et al. [27]. The question marks represent unknown phenomena. ROS, reactive oxygen species. ROOH, alkyl hydroperoxide; ·OR, alkoxy radical; H_2_O_2_, hydrogen peroxide; O_2_^−^, superoxide radical; OHPs, organic peroxides; ·OH, hydroxyl radical; ONOO^−^, reactive nitrogen radical; ETC, electron transport chain.

Collectively, we proposed the following mechanistic model for Cu^2+^/Ni^2+^/Zn^2+^-modulated AesR derepression (Figure 8). Our findings establish AesR as a central metal-responsive regulator that integrates diverse environmental signals—particularly those perturbing metal homeostasis—to coordinate bacterial defense against oxidative stress, antibiotics, and toxic compounds. This work advances the understanding of MarR family regulation and uncovers a previously unrecognized metal-ion-mediated regulatory cascade, potentially representing a conserved stress adaptation strategy within *Corynebacteriales*. Future studies should explore whether analogous mechanisms exist in other bacterial orders and examine the structural basis of metal-sensing by AesR.

## Data Availability

The original contributions presented in this study are included in the article. Further inquiries can be directed to the corresponding author(s).

## Supplementary Materials

The following supporting information can be downloaded at: https://www.mdpi.com/article/10.3390/microorganisms14071416/s1, Supplementary Methods (Strain construction; Plasmid construction; Fluorescence Dye-Based intracellular ROS Detection; Determination of intracellular ion content) [36]. Supplementary Tables (Table S1: Bacterial strains and plasmids used in this study [37,38,39]; Table S2: Primers used in this study; Table S3: Genome-wide comparison of mRNA levels in *C. glutamicum aesR* mutant (Δ*aesR*) and *C. glutamicum* RES167 parental strain (WT) using RNA-seq analysis.). Supplementary Figures (Figure S1: Comparison of the organization of the *ncgl0018*(*dsbA*)*-ncgl0017*(*ccdA*) and *ncgl0019*(*aesR*)*-ncgl0020* operons genome locus in *C. glutamicum* and related species; Figure S2: Members of the genes-controlling AesR involved in antibiotic resistance (A), stress response (B), degradation of aromatic compounds (C), and cell wall/membrane/envelope biogenesis (D); Figure S3: AesR was required for optimal growth under some stress; Figure S4: The regulatory regions of *ncgl001*8-*ncgl0017* and *aesR*-*ncgl0020* operons; Figure S5: Assays for the *ncgl0018-ncgl0017* or *aesR*-*ncgl0020* co-transcription by reverse transcription PCR; Figure S6: 89 bp *aesR* transcript (corresponding to nucleotides +1 to +89 relative to the translational start codon (ATG) of *aesR gene*) was amplified from the remaining *aesR* ORF in Δ*aesR* mutant with primers Q_aesR_-F and Q_aesR_-R; Figure S7: The NCgl0018 and NCgl0020 were examined in *C. glutamicum*; Figure S8: Mutation in the identified AesR binding site derepressed the expression of the *ncgl0018*-*ncgl0017* and the *aesR*-*ncgl0020* operons; Figure S9: Purification of AesR; Figure S10: Sequence of the *aesR* promoter region of *C. glutamicum* aligned to putative *aesR* promoter regions from other *Corynebacterium* species; Figure S11: Analysis the effect of Cu^1+^ on the AesR binding in the presence of oxidants; Figure S12: Expression of the *ncgl0018*-*ncgl0017* and *aesR*-*ncgl0020* operons was induced by β-lactam antibiotics in an AesR-dependent manner.).
