# Supplementary material for: Regulation of Aromatic Compounds and Environmental Stimuli Response by the MarR Family Regulator AesR in Corynebacterium glutamicum
Source: Microorganisms. 2026 Jun 28;14(7):1416. doi: 10.3390/microorganisms14071416 (PMC13413800; doi:10.3390/microorganisms14071416)
Supplement: Supplementary file 1 [file microorganisms-14-01416-s001.zip › microorganisms-4365096-supplementary.pdf]

Supplemental Data

Supplemental Methods:

Strain construction

The *C. glutamicum* RES167 strains were transformed with pK18mob*sacB*-Δ*aesR* or pK18mob*sacB*-Δ*ncgl0020* by electroporation [1]. 25 μg/ml kanamycin (Kan)-resistant colonies resulting from a single cross-over were isolated and replica-plated on LB supplemented with 20% (w/v) sucrose. Sucrose-resistant colonies resulting from a second cross-over were isolated and deletion of *aesR* or *ncgl0020* was confirmed using colony-PCR.

For constructing chromosomal fusion reporter strains, the *lacZY* fusion reporter plasmids pK18mob*sacB*-$P_{ncgl0018\text{-}ncgl0017}$::*lacZY*, pK18mob*sacB*-$P_{aesR\text{-}ncgl0020}$::*lacZY*, pK18mob*sacB*-$P_{aesR\text{-}ncgl0020M}$::*lacZY*, and pK18mob*sacB*-$P_{ncgl0018\text{-}ncgl0017M}$::*lacZY* were transformed into relevant *C. glutamicum* strains (WT(pXMJ19), Δ*aesR*(pXMJ19), or Δ*aesR*(pXMJ19-*aesR*) by electroporation, respectively. The chromosomal fusion reporter strains were selected on LB agar plates supplemented with 25 μg/ml Kan, 40 μg/ml Nal and 5 μg/ml Chl, and confirmed by colony-PCR.

Plasmid construction

The pK18mob*sacB*-Δ*aesR* plasmids were obtained as follows: the 845-bp upstream and 812-bp downstream regions of *aesR* were amplified from *C. glutamicum* RES167 genomic DNA using the primer pairs DaesR-F1/DaesR-R1 and DaesR-F2/DaesR-R2 listed in Table S2, respectively. Then these two products were fused by SOE-PCR with the primer pairs DaesR-F1/DaesR-R2, ligated into the *Eco*RI/*Bam*HI sites of pK18*mobsacB*, and directly used to transform *E. coli* JM109 to yield the pK18*mobsacB*-Δ*aesR* plasmid. A similar strategy was used to generate pK18mob*sacB*-Δ*ncgl0020*.

The pK18mob*sacB*-$P_{ncgl0018\text{-}ncgl0017}$::*lacZY* and pK18mob*sacB*-$P_{aesR\text{-}ncgl0020}$::*lacZY* recombinant plasmids were obtained as follows: Firstly, the primers $P_{ncgl0018\text{-}ncgl0017}$-F/$P_{ncgl0018\text{-}ncgl0017}$-R and lacZY-F1/lacZY-R were used in the first round of PCR to amplify the promoter DNA fragments of the *ncgl0018-ncgl0017* operon (corresponding to nucleotides +15 to -700 relative to the translational start codon (ATG) of *ncgl0018*) and the *lacZY* DNA fragments, respectively. Secondly, $P_{ncgl0018\text{-}ncgl0017}$-F/lacZY-R as primer pair and the first round PCR products as templates were used to perform the second round of PCR, and the resulting fragments were digested with *Sma*I and *Pst*I, and inserted into *Sma*I/*Pst*I restricted pK18mob*sacB* suicide vector to obtain the pK18mob*sacB*-$P_{ncgl0018\text{-}ncgl0017}$::*lacZY* [2]. Similarly, the DNA promoter sequences of the *aesR-ncgl0020* operon ($P_{aesR\text{-}ncgl0020}$, +15 to -600 bp corresponding to the translational start codon (ATG) of *aesR*) were amplified. The resulting fragments were fused with the *lacZY* fragments, digested and inserted into *Sma*I/*Pst*I restricted pK18mob*sacB* suicide vector, obtaining pK18mob*sacB*-$P_{aesR\text{-}ncgl0020}$::*lacZY*.

pK18mob*sacB*-$P_{aesR\text{-}ncgl0020M}$::*lacZY* and pK18mob*sacB*-$P_{ncgl0018\text{-}ncgl0017M}$::*lacZY* were obtained as described [3]. First, 615-bp promoter DNA of the *aesR-ncgl0020* operon containing the mutation in the DNA sequence-protecting AesR ($P_{aesR\text{-}ncgl0020M}$) was constructed by means of overlap SOE-PCR-based directed mutagenesis. Primer pairs $P_{aesR\text{-}ncgl0020}$-F/Binding site mutant-R and Binding site mutant-F/O_{aesR}-R were used to amplify segments 1 and 2, respectively. The second round of PCR was carried out using $P_{aesR\text{-}ncgl0020}$-F/$P_{aesR\text{-}ncgl0020}$-R as primer pair and segments 1 and 2 as templates to obtain $P_{aesR\text{-}ncgl0020M}$. Then, the resulting 615-bp $P_{aesR\text{-}ncgl0020M}$ DNA fragment was fused to a *lacZY* reporter gene using $P_{aesR\text{-}ncgl0020}$-F/lacZY-R as primers. Finally, $P_{aesR\text{-}ncgl0020M}$::*lacZY* DNA fragment was digested with *Sma*I and *Pst*I and inserted into pK18*mobsacB* to generate pK18mob*sacB*-$P_{aesR\text{-}ncgl0020M}$::*lacZY*. pk18mob*sacB*-$P_{ncgl0018\text{-}ncgl0017M}$::*lacZY* plasmid was constructed with similar methods.

Fluorescence Dye-Based intracellular ROS Detection

To detect intracellular ROS, the fluorescent reporter dye 2′, 7′-dichlorodihydrofluorescein diacetate (H2DCFDA, Invitrogen) was used, as previously described [3]. Briefly, 1 ml samples were collected after treatment and then resuspended in 1 ml of PBS containing 10 μM H2DCFDA. Samples were incubated in dark for 20 min. The cells were then pelleted, the supernatant removed, and were resuspended in 1 ml filtere-sterilized PBS. Two hundred microliters of the resultant cell suspension were transferred to a dark 96-well plate. Fluorescence signals were measured using a SpectraMax M2 Plate Reader (Molecular Devices) with excitation/emission wavelengths of 495/520 nm. The results shown represented the mean of one representative assay performed in triplicate, and error bars represent standard deviation. Statistical analysis was carried out with Student's *t*-test.

Determination of intracellular ion content

Intracellular ion content was determined as described previously [4]. Briefly, *C. glutamicum* strains were grown in LB until stationary phase. After 20 ml culture solutions were collected and washed with PBS for two times, the pellets were re-dissolved in 20 ml PBS buffer containing 1% glucose and xenobiotics (1.5 mg/ml PEN, 2 μg/ml ERY, 5.5 mM CHP, or 35 mM *t*-BHP), and then incubated further for 30 min. These cultures were centrifuged at 4000 rpm for 10 min. The wet cell pellet weight was measured and bacteria were chemically lysed

using Bugbuster (Novagen, Madison, WI) according to the manufacturer's instructions. Bacteria were resuspended in Bugbuster solution by pipetting and incubation on a rotating mixer at a slow setting for 60 min. Total protein for each sample was measured by using NanoDrop ND-1000 spectrophotometer (NanoDrop Technologies) according to the manufacturer's instructions. Each sample was diluted 100-fold in 2% molecular grade nitric acid to a total volume of 10 ml. Samples were analyzed by Inductively coupled plasma mass spectrometry (ICP-MS) (Varian 802-MS), and the results were corrected using the appropriate buffers for reference and dilution factors. Triplicate cultures of each strain were analyzed during a single experiment and the experiment was repeated at least three times.

1. Shen, X.H.; Jiang, C.Y.; Huang, Y.; Liu, Z.P.; Liu, S.J. Functional identification of novel genes involved in the glutathione-independent gentisate pathway in *Corynebacterium glutamicum*. *Appl Environ Microbiol.* **2005**,71(7), 3442-3452.

2. Si, M.; Wang, J.; Xiao, X.; Guan, J. Zhang, Y.; Ding, W.; Chaudhry, M.T.; Wang, Y.; Shen, X. Ohr Protects *Corynebacterium glutamicum* against Organic Hydroperoxide Induced Oxidative Stress. *PLoS One.* **2015**,10(6):e0131634.

3. Si, M.; Chen, C.; Su, T.; Che, C.; Yao, S.; Liang, G.; Li, G.; Yang, G. CosR is an oxidative stress sensing a MarR-type transcriptional repressor in *Corynebacterium glutamicum*. *Biochem J.* **2018**,475: 3979-3995.

4. Si, M.; Wang, Y.; Zhang, B.; Zhao, C.; Kang, Y.; Bai, H.; Wei, D.; Zhu, L.; Zhang, L.; Dong, T.G.; Shen, X. Manganese scavenging and oxidative stress response mediated by type VI secretion system in *Burkholderia thailandensis*. *Proc Natl Acad Sci USA*. **2017**,114(11):E2233-E2242.

Supplemental Tables:

Table S1. Bacterial strains and plasmids used in this study.

Strains or plasmids	Relevant genotype description	References
Strains		
Corynebacterium glutamicum		
RES167	Restriction-deficient mutant of ATCC13032, Δ(*cglIM-cglIR-cglIIR*)	[5]
Δ*aesR*	*aesR* deleted in RES167	This study
WT(pXMJ19)	wild type RES167 containing pXMJ19 vector	This study
Δ*aesR*(pXMJ19)	Δ*aesR* containing pXMJ19 vector	This study
Δ*aesR*(pXMJ19-*aesR*)	Δ*aesR* containing pXMJ19-*aesR* vector	This study
Δ*ncgl0018*		[6]
Δ*ncgl0020*	*ncgl0020* deleted in RES167	This study
E. coli		
BL21(DE3)	Host for expression vector pET28a	Novagen
JM109	recA1 supE44 endA1 hsdR17 gyrA96 relA1 thi Δ(lac-proAB)F′(traD36 proABlacI^q lacΔZM15)	Stratagene(catalog no. 200235)
Plasmids		
pK18mob*sacB*	Suicide plasmid carrying *sacB* for selecting double crossover in *C. glutamicum*, Km^r	[7]
pK18mob*sacB*-Δ*aesR*	Construct used for in-frame deletion of *aesR*	This study
pK18mobsacB-Δ*ncgl0020*	Construct used for in-frame deletion of *ncgl0020*	This study
pK18mob*sacB*-$P_{aesR\text{-}ncgl0020}$::*lacZY*	$P_{aesR\text{-}ncgl0020}$::*lacZY* fusion in pK18mob*sacB*	This study

pK18mob*sacB*-$P_{ncgl0018-ncgl0017}$::*lacZY*	$P_{ncgl0018-ncgl0017}$::*lacZY* fusion in pK18mob*sacB*	This study
pXMJ19	Shuttle vector (Ptac lacI^{q} pBL1 oriV$_{C.\ glutamicum}$ pK18 oriV$_{E.\ coli}$)	[8]
pXMJ19-*aesR*	*aesR* cloned into pXMJ19 for complementation	This study
pET28a	Expression vector with N-terminal hexahistidine affinity tag	Novagen
pET28a-*aesR*	*aesR* in pET28a	This study
pET28a-*ncgl0018*	*ncgl0018* in pET28a	[6]
pET28a-*ncgl0020*	*ncgl0020* in pET28a	This study
pET28a-SUMO	Expression vector with N-terminal hexahistidine affinity tag and SUMO	This study
pET28a-SUMO-*aesR*	*aesR* in pET28a-SUMO	This study

References:

5. Tauch, A.; Kirchner, O.; Löffler, B.; Götker, S.; Pühler, A.; Kalinowski, J. Efficient electrotransformation of *Corynebacterium diphtheriae* with a mini-replicon derived from the *Corynebacterium glutamicum* plasmid pGA1. *Curr. Microbiol.* **2002**,45: 362-367.

6. Chen, K.; Yu, X.; Zhang, X.; Li, X.; Liu, Y.; Si, M.; Su, T. Involvement of a mycothiol-dependent reductase NCgl0018 in oxidative stress response of C*orynebacterium glutamicum*. *J Gen Appl Microbiol.* **2021**,67(6): 225-239.

7. Karimova, G.; Pidoux, J.; Ullmann, A.; Ladant, D. A bacterial two-hybrid system based on a reconstituted signal transduction pathway. *Proc Natl Acad Sci USA.* **1998**,95: 5752-5756.

8. Schäfer, A.; Tauch, A.; Jäger, W.; Kalinowski, J.; Thierbach, J.; Pühler, A. Small mobilizable multi-purpose cloning vectors derived from the *Escherichia coli* plasmids pK18 and pK19: selection of defined deletions in the chromosome of *Corynebacterium glutamicum*. *Gene.* **1994**,145: 69-73.

Table S2. Primers used in this study.

Primers	5'-3' sequence	
C$_{aesR}$-F	CGCGGATCCGACTCTAGAGGATCCAAAGGAGGACAACC ATGAGCATCGAGCCAGGAATC(*Bam*HI)	For cloning *aesR* pXMJ19
C$_{aesR}$-R	TCGCCCGGGTCATTTCCTACCCTCCTGCTGT(*Sma*I)	
O$_{aesR}$-F	ATGGGTCGCGGATCCGAATTCATGAGCATCGAGCCAGGAATC(*Eco*RI)	For cloning *aesR* into pET28a
O$_{aesR}$-R	GTGGTGGTGGTGGTGCTCGAGTCATTTCCTACCCTCCTGCTGT(*Xho*I)	
O$_{aesR}$-F1	AGAGAACAGATTGGTGGATCCATGAGCATCGAGCCAGGAATC(*Bam*HI)	For cloning *atsR* into pET28a-SUMO
O$_{aesR}$-R1	GTGGTGGTGGTGGTGCTCGAGTCATTTCCTACCCTCCTGCTGT(*Xho*I)	
D$_{aesR}$-F1	CTATGACATGATTACGAATTCCTTAATAAGTCCCTCTTCGCTGAGC(*Eco*RI)	To generate pK18*mobsacB*-Δ*aesR*
D$_{aesR}$-R1	GCTCGCGATCGGACTGTCAATTTTCCGTG	
D$_{aesR}$-F2	TGACAGTCCGATCGCGAGCTGCTTCTGGA	
D$_{aesR}$-R2	CAGGTCGACTCTAGAGGATCCAAAGAGCCCGAACCAACGC(*Bam*HI)	
D$_{ncgl0020}$-F1	CGCGGATCCGTATCACTCGCATTTCCTCGGTAC(*Bam*HI)	To generate pK18*mobsacB*-Δ*ncgl0020*
D$_{ncgl0020}$-R1	GCGCAGGACCAAAAATTCCAATGAC	
D$_{ncgl0020}$-F2	GTCATTGGAATTTTTGGTCCTGCGCCTGTTGGGATTTGTCAGCGTTATTG	
D$_{ncgl0020}$-R2	CTAGTCTAGACCGAGATAATCAAAATCAGCAAGG (*Xba*I)	
O$_{ncgl0018}$-F	CGCGGATCCATGACTGCGGCTTCGGCGTTAAC(*Bam*HI)	For cloning *ncgl0018* into pET28a
O$_{ncgl0018}$-R	CCGCTCGAGTTATTCTTTCGCTGCCAGTGCTTGG(*Xho*I)	
O$_{ncgl0020}$-F	CGCGGATCCATGACTGCGGCTTCGGCGTTAAC(*Bam*HI)	For cloning *ncgl0020* into pET28a
O$_{ncgl0020}$-R	ACGCGTCGACTTACGGCAGGGAACACCCGGTTAG(*Sal*I)	
P$_{ncgl0019-ncgl0020}$-F	TCCCCCGGGCCATTCAATGCGGAGGTTTCCATCTTC(*Sma*I)	To generate pK18mob*sacB*-$P_{aesR-ncgl0020}$::*lacZY*
P$_{ncgl0019-ncgl0020}$-R	TGGCTCGATGCTCATGCTTCACTTCC	
lacZY-F1	GGAAGTGAAGCATGAGCATCGAGCCAACTAGTACTAGTATGACCATGATTA CGGATTC(*Spe*I)	

lacZY-R	AAAACTGCAGTTAAGCGACTTCATTCACCTG(*Pst*I)	
$P_{ncgl0018-ncgl0017}$-F	TCCCCCGGGGCGCTGGCGCTGCGTGGCGAAG(*Sma*I)	To generate pK18mob*sacB*-$P_{ncgl0018-ncgl0017}$::*lacZY*
$P_{ncgl0018-ncgl0017}$-R	ATTCTCCTTAACCATCCCTGCTGGG	
lacZY-F2	CCCAGCAGGGATGGTTAAGGAGAATACTAGTACTAGTATGACCATGATTACGGATTC(*Spe*I)	
lacZY-R	AAAACTGCAGTTAAGCGACTTCATTCACCTG (*Pst*I)	
Binding site mutant-F	GATACCGCTATAATCCTGTATCAGCTGATGGTCAGTGATGTTATGTATTTDATTGAAGTTGAACTGTG	To generate mutated promoter DNA segment and perform *lacZY*
Binding site mutant-R	CATAACATCACTGACCATCAGCTGATACAGGATTATAGCGGTATCTTGAGTTTTATTCATGGCTTTTAG	
QaesR-F	ATGAGCATCGAGCCAGGAATC	RT-PCR
QaesR-R	CGGACTGTCAATTTTCCGTG	
Qncgl0020-F	CATGAAGCTTATCGACGCC	RT-PCR
Qncgl0020-R	CATGGGCAAGGACTGCCTC	
Qncgl0018-F	CGTTTCAGCCGCTGCGACTTCG	RT-PCR
Qncgl0018-R	ATTGGTAGTCGGAAAACACCAC	
Qncgl0017-F	ATCGTGCTGATTATCCTAGGTT	RT-PCR
Qncgl0017-R	CGGAACAAAAACCGGCAATAC	
Qncgl0014-F	GCTTGTGGCTTTTCTATTTGTGG	RT-PCR
Qncgl0014-R	CCCACCGATTTCAGGGGATAAC	
Qncgl0039-F	AACCGCACCCCGGTTAAGATC	RT-PCR
Qncgl0039-R	GCCGATGCCATAGTTCCATGC	
Qncgl0115-F	GCCATGCCTATCGACTTCCTCC	RT-PCR
Qncgl0115-R	AGCCTTGGGGTTTTGCCACGG	
Qncgl0313-F	GTCGGCGATTTCGTCATCTTG	RT-PCR
Qncgl0313-R	CGCGCCAATACCCAGTGCTG	
Qncgl0522-F	GCAACATCCCCCGGTACCCGG	RT-PCR
Qncgl0522-R	GTGAGTGCTGCGGTAATGATG	
Qncgl0807-F	ATCACTAAAGATAGTGTTCCAG	RT-PCR
Qncgl0807-R	CTCCCGCAAATGTCGCGGAG	
Qncgl1009-F	GAATTTATTCGGACGAGTCCAG	RT-PCR
Qncgl1009-R	CCGCAAACTGCCCACGAAGAAC	
Qncgl1283-F	AGCGCATAGTCGGAGCTGTTC	RT-PCR
Qncgl1283-R	CACAAAGAACTCTAACCATTC	
Qncgl1645-F	TTGGAAGACTGCATTAATTATC	RT-PCR
Qncgl1645-R	CGAAAGTCAAATTCTCTTTGAG	
Qncgl2013-F	GACGAGGAAGGGGCAGATGAGC	RT-PCR
Qncgl2013-R	CAGCAATTGATCAACATCTTCTTC	
Qncgl2502-F	CAGTATGAAGGGCTTCAAAAAC	RT-PCR
Qncgl2502-R	GCGGGAAGGTGACGTCGTACTG	
Qncgl2550-F	TTTTCTGACGGCAAATATGG	RT-PCR
Qncgl2550-R	CCACAAAATCCGGCACCGCC	
Qncgl2728-F	GTGATTACTGGTGAAAATGGG	RT-PCR
QNCgl2728-R	CTCTCCGCACCAACTTCAACG	
Qncgl2950-F	ACACACTTTGGAGTCCATTC	RT-PCR
Qncgl2950-R	CCGATTGGCTCAACCTCTGC	
Qncgl2421-F	CAACAACTTCCAGGTCACTAC	RT-PCR
Qncgl2421-R	CGACATCGTGGGGCCAGAAGC	
ncgl0018-F	CCCTGCCACAGATGATGAAGCATG<br>CATG<br>GGCATCGGCATC	To produce 1000 bp F1
ncgl0017-R	TGCCTGCCCAAGAAGGTGAATTCC	
EaesR-F	GGAGCAATACACCGCCGGCATC	To produce 370 bp F2

	GGCATCGGCATC	
ncgl0020-R	TTTGTGGAAGAAGCGCTGGACC	
EaesR-F	CCTGCTGGGAAGCCCTCAACTAAAAG	To produce 220 bp EMSA *aesR* promoter DNA
EaesR-R	GGCTCGATGCTCATGCTTCACTTCC	
Control-F	CCTGCGTACACAACGATCGCAACC	To produce 221 bp control EMSA DNA
Control-R	AGCAGCTCGCGATCAGTTGCCGTG	

Underlined sites indicated restriction enzyme cutting sites added for cloning. Letters in italic denoted the mutation sites in overlap PCR for site-directed mutation.

Table S3 Genome-wide comparison of mRNA levels in *C. glutamicum* aesR mutant (Δ*aesR*) and *C. glutamicum* RES167 parental strain (WT) using RNA-seq analysis.

Accession	Gene name	Predicted function	Log$_2$^{Ratio}	*p*-value [b]
Genes with a decreased mRNA level in Δ*aesR* mutant				
NCgl0002		DNA polymerase III subunit beta	-1.99	0.000432
NCgl0006		Hypothetical protein	-1.38	0.001884
NCgl0007		Membrane protein	-1.47	0.000361
NCgl0008		Hypothetical protein	-1.48	0.001315
NCgl0014		Uncharacterized membrane protein	-1.38	0.000755
NCgl0015		LysR family transcriptional regulator	-1.50	0.000135
NCgl0023	*ohr*	Stress-induced protein	-1.45	0.002268
NCgl0033		Peptidyl-prolyl cis-trans isomerase (rotamase) - cyclophilin family	-1.65	0.000345
NCgl0035		AraC family transcriptional regulator	-1.32	0.000769
NCgl0036		ABC-type transporter, permease components	-1.44	0.000179
NCgl0039		Septation inhibitor protein	-2.24	1.30E-09
NCgl0047		Hypothetical protein	-1.41	0.001366
NCgl0048		Hypothetical protein	-2.09	4.40E-08
NCgl0051		Hydroxyacylglutathione hydrolase	-1.75	8.70E-07
NCgl0052		Hypothetical membrane protein	-1.64	5.89E-05
NCgl0053		Rhodanese-related sulfurtransferases	-2.09	8.48E-08
NCgl0054		Rhodanese-related sulfurtransferases	-1.41	0.000552
NCgl0056		Hypothetical protein	-1.52	0.002198
NCgl0057		Hypothetical protein	-1.47	0.01078
NCgl0058		Membrane protein	-1.27	0.000456
NCgl0059		Hypothetical protein	-1.26	0.00165
NCgl0061		4-oxalocrotonate tautomerase	-1.31	0.004735
NCgl0069		Phosphoglycerate dehydrogenase and related dehydrogenases	-1.33	0.007795
NCgl0070		Hypothetical protein	-1.91	2.96E-06
NCgl0082		Mar family transcriptional regulator	-1.30	0.001643
NCgl0084	*ureB*	Urea amidohydrolase (urease) gamma subunit	-1.61	9.41E-06
NCgl0087	*ureF*	Urease accessory protein	-1.59	0.000299
NCgl0106		Lactoylglutathione lyase and related lyases	-2.11	1.06E-07
NCgl0107	*sixA*	Phosphohistidine phosphatase	-1.34	0.004488
NCgl0114		Uncharacterized BCR	-1.63	1.76E-05

NCgl0115		3-methyladenine DNA glycosylase	-1.65	0.000146
NCgl0116		Hypothetical protein	-1.50	0.000894
NCgl0130		Chloramphenicol-resistant protein	-2.26	1.93E-08
NCgl0131		Predicted membrane protein	-1.25	0.009402
NCgl0138		Hypothetical protein	-1.87	9.77E-06
NCgl0142		3-methyladenine DNA glycosylase	-1.85	6.54E-05
NCgl0140		Acetyltransferases (the isoleucine patch superfamily)	-1.25	0.004674
NCgl0141		Alkylated DNA repair protein	-1.22	0.003191
NCgl0149		Putative translation initiation inhibitor	-2.26	3.19E-09
NCgl0170		Hypothetical protein	-1.41	0.0058
NCgl0172		Hypothetical membrane protein	-2.13	3.86E-08
NCgl0173		ArsR family Transcriptional regulators	-1.87	3.27E-05
NCgl0177		Membrane protein	-2.15	2.63E-16
NCgl0179		Transposase	-1.63	2.03E-06
NCgl0188		Hypothetical protein	-1.79	1.13E-06
NCgl0201		Membrane protein	-2.05	9.59E-08
NCgl0204		Hypothetical protein	-3.23	2.10E-22
NCgl0210		ABC-type transporter, permease components	-1.45	0.002111
NCgl0212		Molybdopterin synthase sulfur carrier	-1.34	0.003789
NCgl0216		Hypothetical protein	-1.57	0.000357
NCgl0217		Hypothetical protein	-1.48	0.00064
NCgl0221	*mgtE*	Mg/Co/Ni efflux transporter MgtE (contains CBS domain)	-1.21	0.003678
NCgl0236		Transposase	-1.23	0.009653
NCgl0240	*ebfC*	Nucleoid-associated protein	-1.32	0.00917
NCgl0249		Hypothetical membrane protein	-2.59	3.07E-12
NCgl0254		Predicted branched-chain amino acid permease (azaleucine resistance)	-1.08	0.006651
NCgl0256		Hypothetical membrane protein	-2.05	1.05E-06
NCgl0258	*acr3*	Arsenite efflux pump ACR3 and related permeases	-1.32	0.008538
NCgl0271		Uncharacterized membrane-associated protein	-1.67	0.000966
NCgl0280		MarR family transcriptional regulator	-1.87	1.21E-06
NCgl0287		Hypothetical protein	-1.26	0.00969
NCgl0309		Adenylate kinase and related kinases	-1.40	0.006504
NCgl0310		Pseudouridylate synthases, 23S RNA-specific	-1.86	2.44E-06
NCgl0313		Class III Zn-dependent alcohol dehydrogenase	-2.76	8.58E-11
NCgl0318		Small membrane protein	-1.27	0.006711
NCgl0323		Hypothetical membrane protein	-3.47	1.56E-24
NCgl0329		Periplasmic component of ABC-type Fe^{3+}-siderophore transport system	-2.06	4.85E-07
NCgl0335		Hypothetical membrane protein	-1.63	3.70E-05
NCgl0338		Protein-tyrosine-phosphatase	-1.44	0.000544
NCgl0341		Pyridoxal phosphate-dependent enzyme apparently involved in	-1.29	0.003705
NCgl0342		Sugar transferases involved in lipopolysaccharide synthesis	-2.64	5.42E-22
NCgl0343		Predicted glycosyltransferases	-1.96	3.89E-07
NCgl0344		Membrane protein involved in the export of O-antigen and teichoic acid	-1.58	0.000284
NCgl0345		UDP-N-acetylglucosamine enolpyruvyl transferase	-2.05	1.87E-07
NCgl0346	*murB*	UDP-N-acetylmuramate dehydrogenase	-2.53	8.41E-13
NCgl0347		Glycosyltransferases involved in cell wall biogenesis	-1.63	0.000115
NCgl0349		Hypothetical membrane protein	-2.52	4.67E-12
NCgl0350		Acyltransferases	-2.35	2.09E-11
NCgl0351		Predicted UDP-glucose 6-dehydrogenase	-1.72	1.70E-05

NCgl0352		Hypothetical membrane protein	-3.43	4.81E-21
NCgl0353		Glycosyltransferases involved in cell wall biogenesis	-2.77	1.01E-14
NCgl0354		Acetyltransferases (the isoleucine patch superfamily)	-2.40	5.32E-10
NCgl0365		Hypothetical protein	-2.37	4.47E-08
NCgl0368		TetR family transcriptional regulator	-1.42	0.001121
NCgl0373		Hypothetical protein	-2.73	1.79E-14
NCgl0382		Membrane protein	-1.42	0.001095
NCgl0393		Hypothetical protein	-3.35	1.88E-17
NCgl0399		Excisionase	-1.86	0.002322
NCgl0404		Arsenate reductase	-1.90	2.94E-05
NCgl0413		ABC transporter periplasmic component	-2.70	1.21E-12
NCgl0414		Uroporphyrinogen-III synthase	-1.50	0.003022
NCgl0430		ArsR family transcriptional regulator	-1.94	4.42E-07
NCgl0440		Hypothetical protein	-2.35	5.33E-11
NCgl0461		PucR family transcriptional regulator	-2.02	2.47E-07
NCgl0462		4-aminobutyrate aminotransferase	-1.48	0.000416
NCgl0465		Hypothetical copper-exporting P-type ATPase	-1.37	0.000673
NCgl0485		Acetyl-CoA synthase	-1.56	2.39E-05
NCgl0522		Hypothetical membrane protein	-1.54	0.001
NCgl0531		IclR family transcriptional regulator	-1.59	3.36E-05
NCgl0561		Hypothetical protein	-2.49	3.19E-12
NCgl0562		Hypothetical protein	-1.40	0.003031
NCgl0565		Transport protein	-1.87	9.62E-05
NCgl0577		Hypothetical protein	-1.32	0.005073
NCgl0592		Hypothetical protein	-1.43	0.003617
NCgl0595		C50 carotenoid epsilon cyclase	-1.70	7.82E-06
NCgl0597		Phytoene dehydrogenase and related proteins	-1.93	7.55E-08
NCgl0599		Predicted drug exporters of the RND superfamily	-1.63	9.80E-06
NCgl0600		Geranylgeranyl pyrophosphate synthase	-1.85	5.25E-06
NCgl0601		MarR family transcriptional regulator	-2.02	3.03E-06
NCgl0602		Bacterial lipocalin	-1.90	4.19E-07
NCgl0603		Predicted nucleoside-diphosphate-sugar epimerases	-2.57	8.95E-11
NCgl0605		Glycosyltransferases involved in cell wall biogenesis	-1.97	9.62E-07
NCgl0607		ABC-type transporter, ATPase component	-1.29	0.004481
NCgl0620	*ubiA*	4-hydroxybenzoate polyprenyltransferase	-2.38	2.26E-09
NCgl0643		Hypothetical membrane protein	-2.25	2.47E-22
NCgl0660		Hypothetical protein	-1.28	0.001241
NCgl0661		Uncharacterized protein with SCP/PR1 domains	-1.37	0.00158
NCgl0676		Hypothetical protein	-1.46	0.00241
NCgl0724		Predicted amidophosphoribosyltransferases	-1.41	0.000535
NCgl0735		Hypothetical membrane protein	-1.32	0.001612
NCgl0738		Hypothetical protein	-2.21	1.12E-09
NCgl0747		Predicted metal-dependent hydrolase	-2.15	6.71E-09
NCgl0750		Membrane protein	-1.53	0.002336
NCgl0756		Membrane protein	-1.37	0.005087
NCgl0758		Hypothetical protein	-3.39	4.52E-14
NCgl0759		Hypothetical protein	-3.06	3.46E-14
NCgl0760		Hypothetical protein	-1.89	1.44E-06
NCgl0761		Hypothetical protein	-1.78	2.82E-06

NCgl0763		Secreted protein	-1.94	5.34E-06
NCgl0764		Replicative DNA helicase	-1.54	0.000508
NCgl0780		PLP-dependent aminotransferases	-2.07	2.09E-06
NCgl0789		Hypothetical protein	-1.52	2.90E-05
NCgl0797		Acetyl-CoA carboxylase beta subunit	-2.09	0.000938
NCgl0800		Hypothetical protein	-1.33	0.006136
NCgl0803		Hypothetical protein	-1.87	2.36E-06
NCgl0804		mRNA-binding protein	-1.34	0.003671
NCgl0806		Hypothetical protein	-1.75	3.84E-05
NCgl0807		Hypothetical protein	-4.00	6.02E-14
NCgl0815		Uncharacterized membrane-associated protein	-1.62	0.001276
NCgl0819		Chorismate mutase	-1.56	0.000634
NCgl0821		ABC-type transporter, permease components	-1.35	0.001576
NCgl0836		ArsR family transcriptional regulator	-1.55	0.00175
NCgl0841		Trypsin-like serine proteases, typically periplasmic, contain C-terminal	-1.90	0.000617
NCgl0844		Transposase	-1.76	8.37E-05
NCgl0859		Uncharacterized ACR, predicted metal-dependent hydrolases	-1.42	0.000737
NCgl0860		Double-stranded beta-helix domain	-1.20	0.00055
NCgl0863		Transposase	-1.42	0.001236
NCgl0867		Ribosomal protein S14	-1.74	6.54E-05
NCgl0868		Ribosomal protein L33	-2.92	7.70E-16
NCgl0869		Ribosomal protein L28	-2.66	4.89E-13
NCgl0878		Antibiotic biosynthesis monooxygenase	-1.75	1.35E-05
NCgl0881		Predicted hydrolases of the HAD superfamily	-1.62	5.55E-05
NCgl0886		TetR family transcriptional regulator	-1.49	0.004448
NCgl0890		Hypothetical protein	-2.01	0.000102
NCgl0896		ABC-type transporter, ATPase component	-1.43	0.000134
NCgl0903		Lactoylglutathione lyase	-1.61	0.004557
NCgl0917		Hypothetical protein	-2.47	4.96E-12
NCgl0918		Hypothetical protein	-1.41	0.000144
NCgl0920		Hypothetical protein	-2.62	1.19E-14
NCgl0921		Hypothetical protein	-2.04	6.98E-08
NCgl0922		Methyl-accepting chemotaxis sensory	-2.19	2.79E-06
NCgl0930		Hypothetical protein	-2.04	3.76E-07
NCgl0932		Hypothetical protein	-1.95	1.27E-06
NCgl0936	*ftsB*	Cell division protein	-1.36	0.004281
NCgl0942	*pspC*	Putative stress-responsive transcriptional regulator	-1.87	0.000109
NCgl0950		Phospho-2-dehydro-3-deoxyheptonate aldolase	-2.44	2.33E-11
NCgl0957		Hypothetical protein	-3.91	5.00E-15
NCgl0968		FMN reductase (NADPH)	-1.70	2.73E-05
NCgl0994		Diguanylate cyclase/phosphodiesterase domain 2 (EAL)	-1.40	0.00222
NCgl0995		Glycosyltransferase	-1.28	0.003933
NCgl1002		2'-5' RNA ligase	-2.12	7.86E-08
NCgl1004		4-carboxymuconolactone decarboxylase	-1.47	0.000229
NCgl1005	*ndsE*	Nucleoside-diphosphate-sugar epimerases	-1.92	1.20E-06
NCgl1019		Transcriptional regulator	-1.85	4.29E-05
NCgl1027		Predicted Zn-dependent hydrolases of the beta-lactamase	-1.50	0.001275
NCgl1042		Hypothetical protein	-1.65	0.000139
NCgl1044		Ketopantoate reductase	-1.69	0.000409

NCgl1049		Arsenate reductase and related proteins, glutaredoxin family	-1.92	2.69E-06
NCgl1056		Membrane protein	-1.79	0.000224
NCgl1075	*sigE*	RNA polymerase sigma factor	-1.70	0.000478
NCgl1076	*cseE*	Anti-sigma factor	-1.44	0.008608
NCgl1093	*mfs*	Permeases of the major facilitator superfamily	-1.49	0.000395
NCgl1095		ABC-type antimicrobial peptide transporter	-2.87	1.69E-14
NCgl1106		NTP pyrophosphohydrolases including oxidative damage repair	-1.57	0.000432
NCgl1107		Hypothetical protein	-2.53	9.84E-11
NCgl1122		Short chain fatty acids transporter	-1.79	3.97E-05
NCgl1126		Hypothetical protein	-1.34	0.002116
NCgl1127		cAMP-binding domains-catabolite gene activator and regulatory subunit	-1.78	1.58E-05
NCgl1135		Hypothetical protein	-2.63	1.11E-07
NCgl1146		Hypothetical protein	-2.65	3.91E-10
NCgl1148		Molybdopterin-guanine dinucleotide biosynthesis protein A	-1.58	0.000361
NCgl1149		Molybdopterin biosynthesis enzyme	-1.37	0.003633
NCgl1157		Hypothetical membrane protein	-1.36	0.004228
NCgl1171		ACR	-3.14	3.77E-18
NCgl1180		Hypothetical protein	-2.72	8.24E-10
NCgl1187		Predicted transcriptional regulators	-1.73	3.99E-05
NCgl1208		Acetyltransferases, including N-acetylases of ribosomal proteins	-1.65	0.000822
NCgl1231		Conserved protein/domain typically associated with flavoprotein	-1.57	0.001547
NCgl1238		ABC-type nitrate/sulfonate/taurine/bicarbonate transport systems,	-2.13	7.47E-08
NCgl1249		Hypothetical protein	-1.59	0.001153
NCgl1259		Hypothetical protein	-2.27	3.76E-09
NCgl1267		D-alanine ligase	-1.74	0.000369
NCgl1281		Integrase	-1.26	0.007122
NCgl1282		Hypothetical protein	-2.21	2.77E-09
NCgl1283		Hypothetical protein	-1.99	3.83E-06
NCgl1285		Hypothetical protein	-1.40	0.002489
NCgl1286		Hypothetical protein	-2.63	7.29E-13
NCgl1289		Secreted protein	-1.99	8.68E-07
NCgl1290		Hypothetical protein	-2.02	3.49E-06
NCgl1291		SapC family protein	-2.67	6.43E-14
NCgl1293		AsmA protein	-2.46	4.19E-12
NCgl1294		Hypothetical protein	-2.42	1.02E-09
NCgl1300		Major facilitator superfamily permease	-1.98	0.000122
NCgl1301		XRE family transcriptional regulator	-2.52	3.48E-05
NCgl1302		Aldo/keto reductase	-2.16	0.000101
NCgl1306		Dephospho-CoA kinase	-2.76	9.80E-09
NCgl1313		Hypothetical protein	-1.78	2.49E-05
NCgl1317		HxlR family transcriptional regulator	-1.44	0.00138
NCgl1321		Beta-lactamase	-2.35	1.19E-10
NCgl1328		Hypothetical protein	-1.58	0.001126
NCgl1351		Tetraacyldisaccharide 4'-kinase	-2.25	5.05E-06
NCgl1377		ABC-type multidrug/protein/lipid transport system, ATPase component	-1.45	0.001615
NCgl1380		Monovalent cation/hydrogen antiporter	-1.96	3.34E-07
NCgl1381		Hypothetical protein	-1.55	0.004368
NCgl1408		Hydroxyethylthiazole kinase	-1.62	0.000234
NCgl1414		Arsenite efflux pump ACR3 and related permeases	-2.07	6.56E-07

NCgl1419		Membrane protein	-1.65	0.001046
NCgl1427		Hypothetical protein	-2.55	2.97E-10
NCgl1431		Trypsin-like serine protease	-1.31	0.002794
NCgl1453		Arsenite efflux pump ACR3 and related permeases	-1.43	0.002463
NCgl1454		Protein-tyrosine-phosphatase	-1.83	3.43E-05
NCgl1455		Protein-tyrosine-phosphatase	-2.22	3.47E-09
NCgl1481		Hypothetical protein	-2.00	7.48E-06
NCgl1484		Glutamine amidotransferase	-1.37	0.004327
NCgl1489		Hypothetical protein	-1.70	8.88E-05
NCgl1490		Hypothetical protein	-1.31	0.004659
NCgl1526		Glyceraldehyde-3-phosphate dehydrogenase	-2.08	2.28E-06
NCgl1553		Hypothetical protein	-1.97	4.31E-06
NCgl1555		Hypothetical protein	-1.25	0.001338
NCgl1562		Signal peptidase, cleaves prepilin-like proteins	-2.69	9.92E-11
NCgl1584		Preprotein translocase subunit	-1.23	0.008989
NCgl1586		Metallo-beta-lactamase superfamily protein	-1.50	0.003605
NCgl1589		Hypothetical membrane protein	-2.70	2.49E-13
NCgl1600		Acyl-CoA thioesterase	-1.33	0.004324
NCgl1601		Hypothetical membrane protein	-1.84	2.08E-06
NCgl1611		Hypothetical protein	-1.43	0.002103
NCgl1612	*dedD*	Cell division protein	-1.29	0.007979
NCgl1615		Hypothetical protein	-1.74	4.39E-06
NCgl1623		ABC-type transporter, ATPase component	-2.79	5.01E-15
NCgl1624		ABC-type transporter, permease components	-1.82	2.36E-06
NCgl1625		Hypothetical protein	-2.33	2.09E-10
NCgl1626		Phosphopantothenoylcysteine synthetase/decarboxylase	-1.62	0.000109
NCgl1627		DNA primase/helicase	-2.53	1.76E-12
NCgl1629		Hypothetical protein	-2.56	1.17E-12
NCgl1633		Hypothetical protein	-1.31	0.002951
NCgl1635		Cell surface protein	-2.26	1.34E-08
NCgl1640		Hypothetical protein	-1.21	0.003604
NCgl1641		Hypothetical protein	-1.27	0.003854
NCgl1642		Hypothetical protein	-1.92	3.04E-06
NCgl1644		Hypothetical protein	-1.35	0.005791
NCgl1645		Site-specific recombinases, DNA invertase Pin homologs	-1.50	0.007452
NCgl1646		Secreted hydrolase	-1.66	0.0005
NCgl1647		Sugar ABC transporter	-3.44	2.45E-20
NCgl1649		Short-chain dehydrogenase	-1.48	0.000503
NCgl1650		GntR family transcriptional regulator	-2.09	3.80E-07
NCgl1652		Hypothetical protein	-2.28	2.35E-22
NCgl1654		Hypothetical protein	-1.56	0.000207
NCgl1656		Secreted protein	-1.76	1.17E-05
NCgl1658		Hypothetical protein	-2.56	4.35E-12
NCgl1659		Hypothetical protein	-1.65	0.000225
NCgl1660		Hypothetical protein	-1.61	0.009707
NCgl1661		Hypothetical protein	-2.89	5.39E-15
NCgl1667		Single-stranded DNA-specific exonuclease	-1.57	6.56E-05
NCgl1668		Hypothetical protein	-1.46	0.002136
NCgl1669		Predicted ATPase	-1.54	0.00029

NCgl1672		Hypothetical membrane protein	-1.48	0.001232
NCgl1676		Hypothetical protein	-2.14	1.49E-08
NCgl1685		Hypothetical membrane protein	-1.25	0.012007
NCgl1689		ATPase with chaperone activity, ATP-binding subunit	-4.92	1.37E-15
NCgl1691		Cysteine-rich and transmembrane domain-containing protein WIH2-like	-2.22	2.05E-17
NCgl1695		Ribosomal protein S13	-3.52	3.59E-11
NCgl1699		C6 finger domain protein	-1.70	0.003313
NCgl1726		Membrane protein	-1.96	7.95E-05
NCgl1739		Hypothetical protein	-2.77	6.44E-07
NCgl1740		DNA methyltransferase	-3.34	2.69E-14
NCgl1741		Secreted protein	-2.52	2.40E-11
NCgl1742		Hypothetical protein	-2.44	3.84E-11
NCgl1744		Beta-1,4-xylosyltransferase	-1.65	3.86E-05
NCgl1745		XRE family transcriptional regulator	-1.65	0.00013
NCgl1747		Hypothetical protein	-2.28	3.11E-09
NCgl1748		Periplasmic serine proteases (ClpP class)	-2.61	1.83E-13
NCgl1749		Secreted protein	-2.30	1.01E-08
NCgl1750		Hypothetical protein	-1.78	4.77E-05
NCgl1752		Polysaccharide biosynthesis protein	-3.12	1.62E-14
NCgl1754		Isoniazid inductible gene protein INIA	-1.60	0.000484
NCgl1755		Asparaginase	-1.77	1.21E-05
NCgl1756	*sufD*	Fe-S cluster assembly protein	-2.24	4.31E-09
NCgl1757		NnrS family protein	-3.89	1.50E-15
NCgl1758		Glycosyltransferase	-2.58	2.04E-07
NCgl1759		Fatty acid desaturase	-2.15	2.23E-08
NCgl1763		Acetamidase	-1.85	3.23E-05
NCgl1765	*aglA*	Alpha-glucosidase AglA	-1.77	3.32E-06
NCgl1766		Trehalose corynomycolyl transferase	-1.71	1.55E-05
NCgl1770		Superfamily II DNA or RNA helicase	-1.67	8.23E-05
NCgl1771		Uncharacterized low-complexity proteins	-1.58	0.000224
NCgl1774		Hypothetical protein	-1.66	9.39E-05
NCgl1815		Trehalose corynomycolyl transferase	-1.40	0.003757
NCgl1816		Integrase	-2.04	1.83E-07
NCgl1820		TesB-like acyl-CoA thioesterase	-1.80	0.000196
NCgl1841		DNA helicase	-1.79	4.93E-06
NCgl1854		Two-component sensor histidine kinase	-1.44	0.006295
NCgl1882		Membrane protein	-2.65	1.40E-11
NCgl1883		Hypothetical membrane protein	-1.24	0.001555
NCgl1888		Uncharacterized protein (competence- and mitomycin-induced)	-1.75	4.39E-05
NCgl1942		ABC-type transporter, permease components	-1.60	0.000281
NCgl1945		ABC-type membrane protein	-1.94	1.83E-06
NCgl1953		Predicted rossmann-fold nucleotide-binding protein involved in DNA	-1.62	4.77E-06
NCgl1954		Predicted ATPase with chaperone activity	-1.29	0.000372
NCgl1955		Predicted endonuclease distantly related to archaeal Holliday junction	-1.67	9.04E-05
NCgl1956		Hypothetical protein	-2.24	2.04E-09
NCgl1967		LacI family transcriptional regulator	-2.02	1.75E-08
NCgl1968		Di- and tricarboxylate transporters	-1.71	2.59E-06
NCgl1970		Hypothetical protein	-1.47	0.001774
NCgl1976		Ribosomal protein S15P/S13E	-1.44	0.001052

NCgl1977		Ankyrin repeat proteins	-1.56	0.000684
NCgl1978		ABC-type transporter, permease components	-1.54	0.000183
NCgl1989		LysR family transcriptional regulator	-2.01	2.42E-07
NCgl1991		Hypothetical protein	-1.52	0.001358
NCgl2001		Hypothetical protein	-1.97	4.20E-07
NCgl2007	*c23o*	Predicted ring-cleavage extradiol dioxygenase	-1.54	0.000355
NCgl2013	*hisF*	Imidazole glycerol phosphate synthase subunit	-2.33	0.000521
NCgl2018		Membrane protein	-1.40	0.003576
NCgl2025		TetR family transcriptional regulator	-1.51	0.000377
NCgl2031		ABC-type transporter, ATPase component	-1.70	4.51E-06
NCgl2036		Hypothetical protein	-2.17	2.26E-06
NCgl2037		Maltooligosyl trehalose synthase	-1.32	0.002646
NCgl2039		Hypothetical protein	-2.25	2.09E-07
NCgl2043		Hypothetical protein	-3.21	1.48E-12
NCgl2044		Uncharacterized ACR	-1.49	0.000852
NCgl2046	*ilvA*	Threonine dehydratase	-2.85	0.000166
NCgl2050		Predicted permeases	-3.05	2.79E-19
NCgl2051		Hypothetical protein	-1.48	0.001477
NCgl2052		Predicted Co/Zn efflux family protein	-1.76	8.21E-05
NCgl2054		Diaminopimelate decarboxylase	-1.57	0.000155
NCgl2055		Cysteine synthase	-1.30	0.006571
NCgl2071		Membrane protein	-2.74	7.55E-14
NCgl2090		Hypothetical protein	-1.28	0.00833
NCgl2097		DUF2029 family protein	-1.24	0.003515
NCgl2099		Hypothetical protein	-1.80	5.94E-06
NCgl2104		1-acyl-sn-glycerol-3-phosphate acyltransferase	-1.35	0.009397
NCgl2112	*ctaE*	Cytochrome C oxidase subunit III	-2.14	1.46E-08
NCgl2115	*ctaC*	Subunit II of heme/copper-type cytochrome/quinol oxidasese	-1.65	1.32E-06
NCgl2134		Hypothetical protein	-1.93	1.74E-06
NCgl2140		Hypothetical protein	-2.32	6.97E-10
NCgl2151		Hypothetical protein	-1.78	3.25E-05
NCgl2163		TetR family transcriptional regulator	-1.82	1.42E-05
NCgl2182		Hypothetical protein	-1.72	2.89E-05
NCgl2183		Hypothetical protein	-1.53	0.00098
NCgl2187		Hypothetical protein	-1.77	3.56E-06
NCgl2189		Hypothetical protein	-1.78	1.32E-05
NCgl2197		Hypothetical protein	-1.40	0.002561
NCgl2199		ArsR family transcriptional regulator	-1.55	0.000478
NCgl2200		Zn^{2+} uptake regulation proteins	-1.39	0.012146
NCgl2224		Hypothetical protein	-2.11	1.58E-08
NCgl2230		Choline-glycine betaine transporter	-1.44	0.0029
NCgl2243		Sugar kinases, ribokinase family	-1.49	0.000999
NCgl2265		Predicted multitransmembrane, metal-binding protein	-2.07	4.17E-09
NCgl2285		Pirin-related protein	-2.06	4.01E-09
NCgl2298		TetR/AcrR family transcriptional regulator	-1.66	3.61E-05
NCgl2300	*vanA*	Ring-hydroxylating dioxygenase	-1.52	0.00087
NCgl2330		Hypothetical protein	-1.41	0.003048
NCgl2334		Hypothetical protein	-1.63	1.52E-05
NCgl2343		Ribosomal protein S20	-1.57	0.000716

NCgl2344		Hypothetical protein	-2.27	1.81E-06
NCgl2350		ABC-type transporter, duplicated ATPase component	-1.29	0.000331
NCgl2352		Nicotinic acid mononucleotide adenylyltransferase	-1.68	6.27E-05
NCgl2367		Hypothetical protein	-1.57	0.005435
NCgl2391		Hypothetical protein	-2.17	3.34E-06
NCgl2394		Hypothetical protein	-1.27	0.001427
NCgl2403	*prxQ*	Peroxiredoxin Q	-2.32	3.15E-06
NCgl2416		Hypothetical protein	-3.54	7.21E-15
NCgl2417		Hypothetical protein	-2.05	6.40E-07
NCgl2422		Metal-dependent hydrolases of the beta-lactamase superfamily III	-1.47	0.00275
NCgl2437	*ctaD*	Subunit I of heme/copper-type cytochrome/quinol oxidasese	-1.56	0.000258
NCgl2441		Mn-dependent transcriptional regulator	-1.42	0.000482
NCgl2459		Membrane protein	-1.99	2.45E-06
NCgl2460		Transposase	-2.24	3.22E-09
NCgl2462		Hypothetical membrane protein	-1.26	0.007661
NCgl2466		Hypothetical protein	-1.25	0.000749
NCgl2475		Acetyltransferase	-1.99	1.96E-06
NCgl2487		Histone acetyltransferase HPA2 and related acetyltransferases	-1.69	5.65E-05
NCgl2502	*mpx*	Glutathione peroxidase	-1.36	0.00899
NCgl2512		Diadenosine tetraphosphate (Ap4A) hydrolase and other HIT family	-1.94	7.96E-06
NCgl2527		LysR family transcriptional regulator	-1.84	0.000131
NCgl2531		Hypothetical protein	-1.55	0.001486
NCgl2533	*thrE*	Threonine exporter	-1.34	0.00214
NCgl2534		Hypothetical protein	-1.58	0.001189
NCgl2543		Hypothetical protein	-1.59	0.003518
NCgl2550		IclR family transcriptional regulator	-1.46	0.003802
NCgl2561		Hypothetical protein	-2.81	6.58E-10
NCgl2566		Putative threonine efflux protein	-1.73	2.12E-05
NCgl2568		Hypothetical membrane protein	-2.55	1.31E-08
NCgl2583		Hypothetical protein	-2.46	7.73E-11
NCgl2593		Hypothetical protein	-1.57	0.000161
NCgl2605		Dethiobiotin synthetase	-1.79	1.91E-05
NCgl2613		Hypothetical protein	-2.02	7.55E-06
NCgl2614		Phosphotransferase system IIC components, glucose/maltose/N-acetylglucosamine-specific	-1.84	1.57E-05
NCgl2638		Multisubunit Na+/H+ antiporter	-1.50	9.46E-05
NCgl2642		Hypothetical protein	-1.93	1.66E-06
NCgl2648		Na+/phosphate symporter	-1.44	0.000715
NCgl2649		Epimerase	-2.29	1.09E-14
NCgl2664		Membrane protein	-3.28	5.05E-21
NCgl2666		Hypothetical protein	-1.66	0.000108
NCgl2667		Two-component system, sensory transduction histidine kinases	-1.83	2.01E-06
NCgl2668		Two-component system, response regulators consisting of a CheY-like	-2.44	8.09E-11
NCgl2671		Membrane protein	-1.76	4.89E-05
NCgl2675		Hypothetical protein	-2.37	2.02E-08
NCgl2683		Sodium/glutamate symport carrier protein	-1.68	5.70E-05
NCgl2685		Co/Zn efflux family protein	-1.63	5.43E-05
NCgl2688		Cystathionine beta-lyases/cystathionine gamma-synthases	-1.47	0.000244
NCgl2692		Hypothetical protein	-1.93	2.39E-07

NCgl2693		Hypothetical protein	-1.67	4.17E-05
NCgl2707		Hypothetical protein	-1.58	0.000437
NCgl2710		Hypothetical protein	-1.95	2.15E-05
NCgl2720		Uncharacterized BCR	-1.46	0.006248
NCgl2721		Hypothetical membrane protein	-1.32	0.005855
NCgl2725		Tricarboxylic transport membrane protein	-1.24	0.000957
NCgl2726		Tricarboxylic transport membrane protein	-1.69	5.07E-05
NCgl2728		ABC-type transporter, ATPase component	-2.04	1.19E-06
NCgl2734		ABC-type transporter, permease components	-1.50	0.00043
NCgl2746		ABC-type cobalamin/Fe^{3+}-siderophores transport systems, ATPase components	-2.28	5.10E-08
NCgl2748		Transposase	-1.66	1.15E-06
NCgl2758		Hypothetical protein	-1.52	0.000962
NCgl2765		Phosphoenolpyruvate carboxykinase	-1.49	0.00802
NCgl2766		Hypothetical membrane protein	-2.06	3.80E-08
NCgl2785		Membrane-associated phospholipid phosphatase	-1.52	0.000656
NCgl2786		Membrane protein	-1.76	2.57E-06
NCgl2788		UDP-galactopyranose mutase	-1.39	0.004139
NCgl2796		Hypothetical protein	-1.38	0.004479
NCgl2801		Membrane protein	-1.37	0.000716
NCgl2811		Hypothetical protein	-2.47	9.93E-08
NCgl2846		GntR family transcriptional regulator	-2.02	0.00247
NCgl2859	*copB*	Probable Cu-transporting ATPase transmembrane protein	-1.35	0.002643
NCgl2865	*copO*	Hypothetical multicopper oxidase	-2.33	1.39E-09
NCgl2875		Copper chaperone	-1.91	2.06E-06
NCgl2877		Hypothetical protein	-2.72	1.49E-11
NCgl2886	*malR*	MarR family transcriptional regulator	-1.44	0.001385
NCgl2887	*uspA*	Universal stress proteinn	-2.13	1.39E-09
NCgl2888		Membrane protein	-1.75	5.63E-06
NCgl2892		ABC-type transporter, ATPase component	-1.24	0.003426
NCgl2895		Hypothetical protein	-1.61	0.000514
NCgl2906		Membrane protein	-1.89	0.000129
NCgl2907		Membrane protein	-2.60	5.35E-10
NCgl2910		Hypothetical protein	-1.61	0.000184
NCgl2911		Hypothetical protein	-1.27	0.005721
NCgl2914		Hypothetical protein	-1.43	0.000841
NCgl2916		Uncharacterized BCR	-1.71	1.40E-05
NCgl2926		Hypothetical protein	-3.04	2.30E-15
NCgl2937		Hypothetical protein	-2.35	1.19E-10
NCgl2921	*genR*	IclR family transcriptional regulator	-1.74	0.001761
NCgl2950		TetR family transcriptional regulator	-1.61	0.004485
NCgl2966		Hydroxymethylpyrimidine/phosphomethylpyrimidine kinase	-1.37	0.000308
NCgl2984	*trxR*	Thioredoxin reductase	-1.68	0.004988
NCgl2985	*trx1*	Thioredoxin	-1.39	0.000834
Genes with an increased mRNA level in Δ*aesR* mutant				
NCgl0017		Cytochrome C biogenesis protein	2.32	3.64E-11
NCgl0018		Protein-disulfide isomerase	2.95	8.71E-14
NCgl0020		Zn-dependent protease with chaperone function	1.85	7.42E-11
NCgl2421		Hypothetical protein	1.51	6.88E-12

The mRNA ratio represented mean values from three independent experiments starting from independent cultures. The strains were cultivated in LB medium, and mRNA was isolated in the exponential growth phase. [a] $\mathrm{Log}_2^{\mathrm{Ratio}}$ was defined by $\log_2$ (the gene mRNA expression ratio of the *Corynebacterium glutamicum* Δ*aesR* mutant to *C. glutamicum* RES167 parental strain (WT)). $\mathrm{Log}_2^{\mathrm{Ratio}}$ values of higher than +1.2 or lower than -1.2 (corresponding to mRNA ratio Δ*aesR*/WT of > 2.3 and < 0.435, respectively) were considered to be significant. The table included those genes that showed a >2.3-fold changed mRNA level (increased or decreased) in at least two of the three experiments and that had a *p* value < 0.01. Genes were ordered according to their position on the genome. [b] Significance determined by *p*-value ($p < 0.01$).

Supplemental Figures:

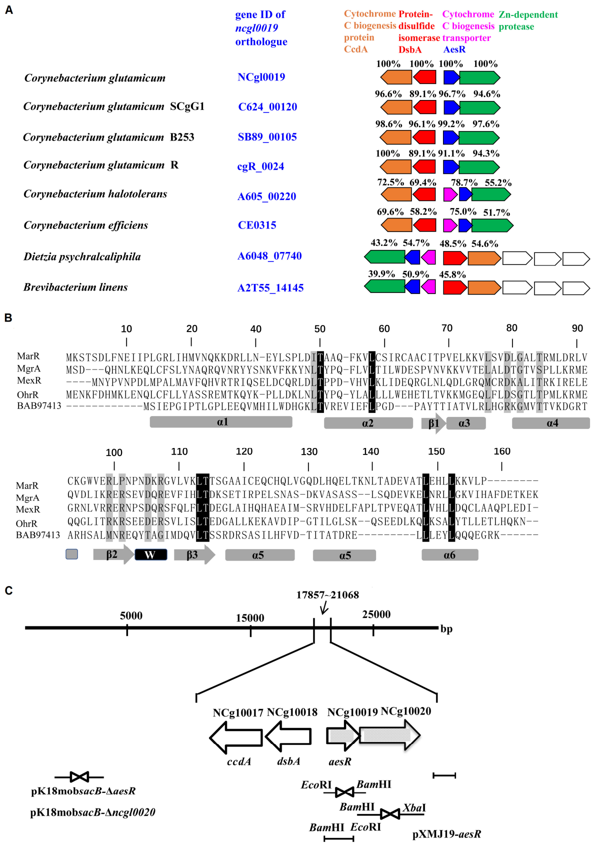

Figure S1 Comparison of the organization of the *ncgl0018-ncgl0017* and *ncgl0019*(*aesR*)*-ncgl0020* operons genome locus in *C. glutamicum* and related species. (A) The percentage identities of the amino acid sequences to the *ncgl0018-ncgl0017* and *aesR-ncgl0020* operons from *C. glutamicum* were taken from NCBI Blast. Amino acid sequence identities to the *C. glutamicum* orthologs were given in the upper column. The genomic contexts of *C. glutamicum dsbA-ccdA* and *aesR-ncgl0020* operons were extracted from microbesonline (http://microbesonline.org). **(B)** Multiple alignment of AesR with representative members of the MarR family. The alignment was generated using ClustalW. Grey and dark shading indicated ≥80% similarity or identity at that position, respectively. Residue numbering was according to the entire alignment. The secondary structure elements predicted using online tools SWISS-MODEL and ESPript 3.0, and indicated below the alignment showed the conservation of the winged helix-turn-helix motif and were based on the MarR crystal structure, with α-helices represented as gray boxes, β-sheets as gray arrows, and the wing as a black box (Alekshun et al., 2001). The proteins used for the alignment: *C. glutamicum* (BAB97413), *Escherichia coli* MarR (EGT68600), *Staphylococcus aureus* MgrA (EES93778), *Pseudomonas aeruginosa* MexR (NP_249115), and *Bacillus subtilis* OhrR (WP_187956060). **(C)** Physical map of the *dsbA-ccdA* and *aesR-ncgl0020* operons in *C. glutamicum* RES167 parental strain (WT) and construction of plasmids for gene disruption (pK18*mobsacB* derivatives) or complementation (pXMJ19 derivatives). Open reading frames (ORFs) were marked by open arrows, and the deleted regions were in grey. The restriction sites were indicated.

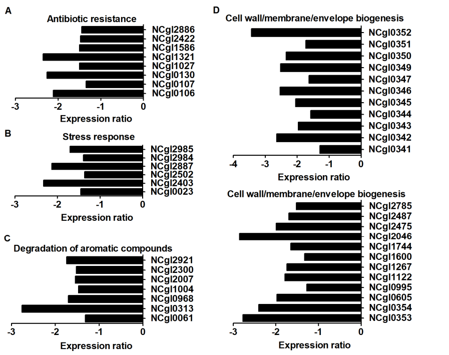

Figure S2 Members of the genes-controlling AesR involved in antibiotic resistance (A), stress response (B), degradation of aromatic compounds (C), and cell wall/membrane/envelope biogenesis (D). Expression ratios for the Δ*aesR* mutant compared to the WT strain were indicated. Gene tags or open reading frames (ORFs) numbers were indicated following the *C. glutamicum* RES167 strain annotation.

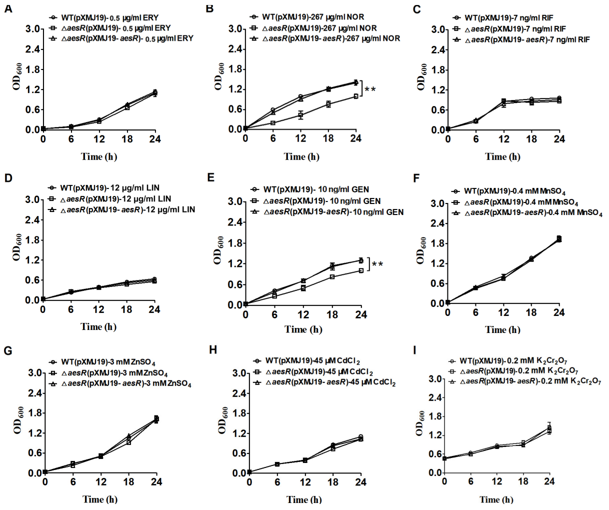

Figure S3 AesR was required for optimal growth under some stress. Growth of indicated strains in LB broth with 100 μg/ml erythromycin (ERY) (**A**), 267 μg/ml norfloxacin (NOR) (**B**), 7 ng/ml rifamycin (RIF) (**C**), 0.5 mg/ml lincomycin (LIN) (**D**), 10 ng/ml gentamycin (GEN) (**E**), 0.4 mM ($MnSO_4$) (**F**), 3 mM hypochlorous acid ($ZnSO_4$) (**G**), 45 μM cadmium chloride ($CdCl_2$) (**H**), or 0.2 mM potassium dichromate ($K_2Cr_2O_7$) (**I**). Data showed the averages of three independent experiments, and error bars indicated the SDs from three independent experiments. **, $P \leq 0.01$.

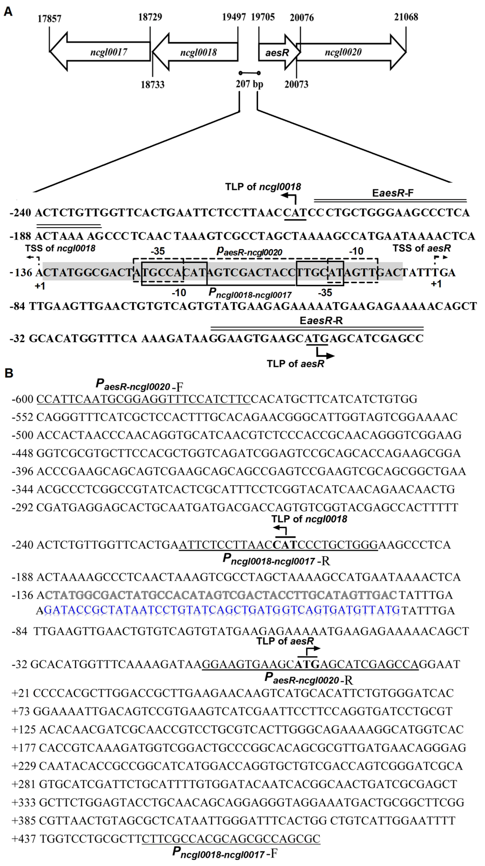

Figure S4 The regulatory regions of *ncgl0018-ncgl0017* and *aesR-ncgl0020* operons. (A) The intergenic region between *ncgl0018* and *aesR* genes was shown in 5'→3' orientation relative to the translational start point (TLP) of the *aesR* gene. The number on the left was relative to the initiation codon ATG (in underlined) of *aesR.* Shaded grey indicated the identified binding site of AesR. The putative transcriptional start sites (+1, TSS) of *ncgl0018* and *aesR* were shown and dotted bent arrows denoted the direction of transcription. The TLPs of *ncgl0018* and *aesR* were indicated with solid bent arrows. The proposed -35 and -10 promoter regions of $P_{ncgl0018\text{-}ncgl0017}$ and $P_{aesR\text{-}ncgl0020}$ were boxed with the solid and dotted lines, respectively. $P_{ncgl0018\text{-}ncgl0017}$ and $P_{aesR\text{-}ncgl0020}$ represented promoters of *ncgl0018-ncgl0017* and *aesR-ncgl0020* operons, respectively. E*aesR*-F/E*aesR*-R, was marked with double underline and used to produce 220 bp EMSA promoter DNA, indicated the location of the primer design. **(B)** The promoter regions were upstream of *ncgl0018-ncgl0017* and *aesR-ncgl0020* operons. Gray shaded indicated the identified binding site of AesR. The TLPs of *ncgl0018* and *aesR* were bold and were shown with solid bent arrows. The sequences used to replace the binding site were shown in blue below the promoter sequences. Promoter fragments for constructing $P_{ncgl0018\text{-}ncgl0017}$*::lacZY* or $P_{aesR\text{-}ncgl0020}$*::lacZY* and analyzing β-galactosidase activities were obtained using primer pair $P_{ncgl0018\text{-}ncgl0017}$-F/$P_{ncgl0018\text{-}ncgl0017}$-R or $P_{aesR\text{-}ncgl0020}$-F/$P_{aesR\text{-}ncgl0020}$-R. The location of the primer design was marked with single underline. The number on the left was relative to the ATG start codon of *aesR.*

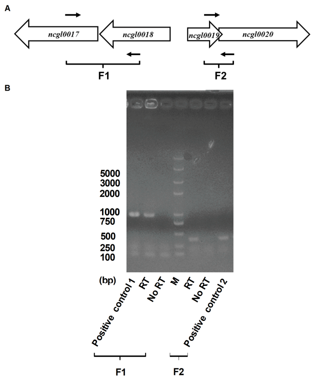

Figure S5 Assays for the *ncgl0018-ncgl0017* or *aesR-ncgl0020* co-transcription by reverse transcription PCR. (A) The operon structure of the *ncgl0018-ncgl0017* or *aesR-ncgl0020* primers those designed for assays and indicated by blank arrows. **(B)** Reverse

transcription PCR assays for the *ncgl0018-ncgl0017* or *aesR-ncgl0020* co-transcription. M, DNA Marker. Negative control PCR reactions omitted the initial reverse transcription step (No-RT). Genomic DNA as template was used as positive control. PCR procedure was as follows: reactions were denatured 95 °C for 50 s, annealed at 58 °C for 40 s, extended at 72 °C for 60 s (*ncgl0018-ncgl0017*) or 25 s (*aesR-ncgl0020*), and repeated 30 cycles.

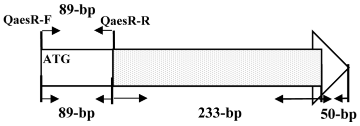

Figure S6 89 bp *aesR* transcript (corresponding to nucleotides +1 to +89 relative to the translational start codon (ATG) of *aesR* gene) was amplified from the remaining *aesR* ORF in Δ*aesR* mutant with primers Q_{aesR}-F and Q_{aesR}-R. *aesR* ORF was marked by open arrow and the deleted region was in grey.

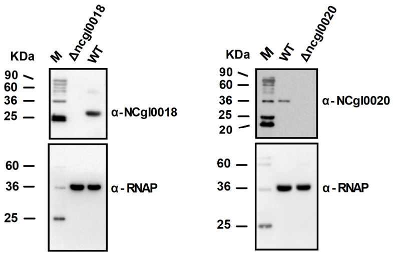

Figure S7 The NCgl0018 and NCgl0020 were examined in *C. glutamicum*. Anti-NCgl0018 and Anti-NCgl0020 antibodies detected a single protein with a mobility consistent with the predicted size that was absent from Δ*ncgl0018 and* Δ*ncgl0020* mutants. Antibody to RNAP was used as a loading control. Data show one representative experiment done in triplicate.

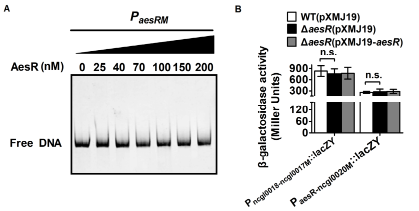

Figure S8 Mutation in the identified AesR binding site derepressed the expression of the *ncgl0018-ncgl0017* and the *aesR-ncgl0020* operons. (A) The interaction between AesR and the 220-bp promoter DNA fragment mutating the identified AesR binding regions (P_{aesRM}). **(B)** β-galactosidase activities were measured in indicated bacterial strains. Relative levels of transcripts were presented as the mean values ± SD calculated from three sets of independent experiments. n.s., not significant.

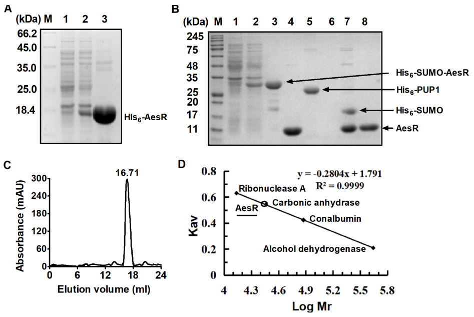

Figure S9 Purification of AesR. (A) Coomassie-stained SDS-PAGE of His_6-AesR. M, broad-range protein marker; lane 1, crude extract of BL21(DE3)(pET28a-*aesR*) strain without IPTG induction; lane 2, crude extract of BL21(DE3)(pET28a-*aesR*) strain with induction; lane 3, purified His_6-AesR protein. **(B)** Coomassie-stained SDS-PAGE of His_6-SUMO-AesR, AesR, and His_6-PUP1. M, broad-range protein marker; lane 1, crude extract of BL21(DE3)(pET28a-SUMO-*aesR*) strain without IPTG induction; lane 2, crude extract of BL21(DE3)(pET28a-SUMO-*aesR*) strain with induction; lane 3, purified His_6-SUMO-AesR protein; lane 4, purified AesR protein; lane 5, purified His_6-PUP1

protein. **(C)** Elution of native AesR from size exclusion column. **(D)** For calibration, a premixed protein molecular mass marker containing the following proteins was used: ribonuclease A (13,700 Da), carbonic anhydrase (29,000 Da), conalbumin (75,000 Da), and alcohol dehydrogenase (150,000 Da) (GE Healthcare, Piscataway, NJ). V_o was determined with blue dextran (2,000 kDa). Native AesR was indicated by clear circle.

	-35 / -10
C. glutamicum	CTATGGCGACT**ATGCCA**CATAGTCGACTACCTTGC**ATAGTT**GA-92-**ATG**
C. halotolerans	ACT**ATGACA**CATAGTCGACTACCTGTC**ATAGTC**GACTACGCGG-29-**ATG**
C. efficiens	ACT**ATGTCA**CATCGTCGACTAAGTTGC**ATAGTC**GACTATGTGG-29-**ATG**
Dietzia psychralcaliphila	GACT**ATTACA**CATAGTCGACTATTGGGG**ATAGTC**GGCCCGTGG-48-**ATG**
Brevibacterium linens	TACT**ATGGAG**CATAGTATACTAATGTGC**ATAGTA**GACTCGTAT-27-**ATG**
**Consensus**	**ACTATGnnnCATAGTCGACTAnnnnnnnTAGTCG**

Figure S10 Sequence of the *aesR* promoter region of *C. glutamicum* aligned to putative *aesR* promoter regions from other *Corynebacterium* species. Indicated were the start of the *aesR* coding region (start codons black bold), putative -10 (underlined and black bold), and the AesR binding site (shaded in gray) for the *C. glutamicum aesR* gene. As shown by the alignment, also the other species possessed putative AesR binding sites in the *aesR* upstream region. The binding sites represented two perfect inverted repeats in five *Corynebacterium* species with the consensus sequence 5′-ACTATGnnnCATAGTCGACTAnnnnnnnTAGTCG-3′.

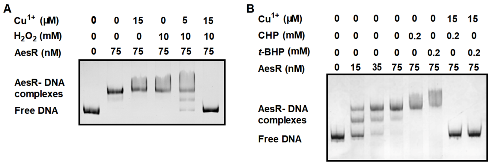

Figure S11 Analysis the effect of Cu^{1+} on the AesR binding in the presence of oxidants. EMSA analysis demonstrating the effect of Cu^{1+} on the DNA binding capability of AesR in the presence of oxidants. Cu^{1+} in the presence of H_2O_2 (**A**), CHP (b), *t*-BHP (**B**) triggered the protein's dissociation from its binding DNA.

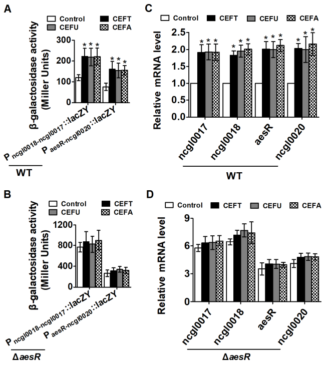

Figure S12 Expression of the *ncgl0018-ncgl0017* and *aesR-ncgl0020* operons was induced by β-lactam antibiotics in an AesR-dependent manner. (**A and B**) β-galactosidase analysis of the promoter activities of the *ncgl0018-ncgl0017* and *aesR-ncgl0020* operons by using the transcriptional $P_{ncgl0018\text{-}ncgl0017}$*::lacZY* and $P_{aesR\text{-}ncgl0020}$*::lacZY* chromosomal fusion reporter expressed in WT and Δ*aesR* strains exposed to various β-lactam antibiotics. (**C and D**) qRT-PCR assay was performed to analyze the expression of the *ncgl0017*, *ncgl0018*, *aesR*, and *ncgl0020* in WT and Δ*aesR* strains exposed to various β-lactam antibiotics. The mRNA levels were presented relative to the value obtained from WT cells without treatment. Relative transcript levels of WT strains without stress treatment were set at a value of 1.0. Data showed the averages of three independent experiments, and error bars indicated the SDs from three independent experiments. Significant difference was analyzed by comparing the expression between the treated and untreated strains.*, $P \leq 0.05$.
